# Making sense of missense variants in *TTN*-related congenital myopathies

**DOI:** 10.1007/s00401-020-02257-0

**Published:** 2021-01-15

**Authors:** Martin Rees, Roksana Nikoopour, Atsushi Fukuzawa, Ay Lin Kho, Miguel A. Fernandez-Garcia, Elizabeth Wraige, Istvan Bodi, Charu Deshpande, Özkan Özdemir, Hülya-Sevcan Daimagüler, Mark Pfuhl, Mark Holt, Birgit Brandmeier, Sarah Grover, Joël Fluss, Cheryl Longman, Maria Elena Farrugia, Emma Matthews, Michael Hanna, Francesco Muntoni, Anna Sarkozy, Rahul Phadke, Ros Quinlivan, Emily C. Oates, Rolf Schröder, Christian Thiel, Jens Reimann, Nicol Voermans, Corrie Erasmus, Erik-Jan Kamsteeg, Chaminda Konersman, Carla Grosmann, Shane McKee, Sandya Tirupathi, Steven A. Moore, Ekkehard Wilichowski, Elke Hobbiebrunken, Gabriele Dekomien, Isabelle Richard, Peter Van den Bergh, Cristina Domínguez-González, Sebahattin Cirak, Ana Ferreiro, Heinz Jungbluth, Mathias Gautel

**Affiliations:** 1 Randall Centre for Cell and Molecular Biophysics, Muscle Biophysics, King’s College London BHF Centre of Research Excellence, London, UK; 2 Department of Paediatric Neurology, Evelina Children’s Hospital, Guy’s & St Thomas’ NHS Foundation Trust, London, UK; 3 Department of Clinical Neuropathology, King’s College Hospital, London, UK; 4 Department of Clinical Genetics, Guy’s Hospital, London, UK; 5 Centre for Molecular Medicine, University of Cologne, Cologne, Germany; 6 Department of Pediatrics, University Hospital Cologne and Faculty of Medicine, University of Cologne, Cologne, Germany; 7 School of Cardiovascular Medicine and Sciences, King’s College London BHF Centre of Research Excellence, London, UK; 8 Pediatric Neurology Unit, Paediatrics Subspecialties Service, Geneva Children’s Hospital, Geneva, Switzerland; 9 West of Scotland Regional Genetics Service, Laboratory Medicine Building, Queen Elizabeth University Hospital, Glasgow, UK; 10 Department of Neurology, Queen Elizabeth University Hospital, Glasgow, UK; 11 MRC Neuromuscular Centre, National Hospital for Neurology and Neurosurgery, Queen’s Square, London, UK; 12 Dubowitz Neuromuscular Centre, Great Ormond Street Hospital for Children, London, UK; 13 NIHR Great Ormond Street Hospital Biomedical Research Centre, Great Ormond Street Institute of Child Health, University College London, Great Ormond Street Hospital Trust, London, UK; 14 School of Biotechnology and Biomolecular Sciences, The University of New South Wales, Sidney, Australia; 15 Kids Neuroscience Centre, Kids Research, The Children’s Hospital at Westmead, Sydney, NSW Australia; 16 Institute of Neuropathology, University Hospital Erlangen, Friedrich-Alexander Universität Erlangen-Nürnberg, Erlangen, Germany; 17 Department of Genetics, University of Erlangen, Erlangen, Germany; 18 Muscle Laboratory, Department of Neurology, University of Bonn Medical Centre, Bonn, Germany; 19 Department of Neurology, Donders Institute for Brain, Cognition and Behaviour, Radboud University, Nijmegen, The Netherlands; 20 Department of Paediatric Neurology, Radboud University, Nijmegen, The Netherlands; 21 Department of Human Genetics, Radboud University Medical Center, Nijmegen, The Netherlands; 22 UCSD, Rady Children’s Hospital, and VA San Diego Healthcare System, San Diego, USA; 23 Gillette Children’s Specialty Care, St Paul, MN USA; 24 Northern Ireland Regional Genetics Service, Belfast City Hospital, Belfast, UK; 25 Department of Paediatric Neurology, Royal Belfast Hospital for Sick Children, Belfast, UK; 26 Department of Pathology, The University of Iowa, Iowa City, IA USA; 27 Department of Paediatric Neurology, University of Göttingen, Göttingen, Germany; 28 Institut Für Humangenetik, Ruhruniversität Bochum, Bochum, Germany; 29 Genethon and UMR_S951, INSERM, Université Evry, Université Paris Saclay, Evry, 91002 Evry, France; 30 Neuromuscular Reference Centre, Department of Neurology, University Hospital Saint-Luc, Brussels, Belgium; 31 Reference Center for Neuromuscular Disorders, Hospital Universitario 12 de Octubre, Madrid, Spain; 32 Centre for Rare Diseases (ZSEK), University of Cologne, Cologne, Germany; 33 Basic and Translational Myology Laboratory, Université de Paris, Paris, France; 34 Centre de Référence Des Maladies Neuromusculaires, APHP, Institut of Myology, GHU Pitié Salpêtrière- Charles Foix, Paris, France; 35 Department of Clinical and Basic Neuroscience, IoPPN, King’s College London, London, UK

## Abstract

Mutations in the sarcomeric protein titin, encoded by *TTN*, are emerging as a common cause of myopathies. The diagnosis of a *TTN*-related myopathy is, however, often not straightforward due to clinico-pathological overlap with other myopathies and the prevalence of *TTN* variants in control populations. Here, we present a combined clinico-pathological, genetic and biophysical approach to the diagnosis of *TTN*-related myopathies and the pathogenicity ascertainment of *TTN* missense variants. We identified 30 patients with a primary *TTN*-related congenital myopathy (CM) and two truncating variants, or one truncating and one missense *TTN* variant, or homozygous for one *TTN* missense variant. We found that *TTN*-related myopathies show considerable overlap with other myopathies but are strongly suggested by a combination of certain clinico-pathological features. Presentation was typically at birth with the clinical course characterized by variable progression of weakness, contractures, scoliosis and respiratory symptoms but sparing of extraocular muscles. Cardiac involvement depended on the variant position. Our biophysical analyses demonstrated that missense mutations associated with CMs are strongly destabilizing and exert their effect when expressed on a truncating background or in homozygosity. We hypothesise that destabilizing *TTN* missense mutations phenocopy truncating variants and are a key pathogenic feature of recessive titinopathies that might be amenable to therapeutic intervention.

**Supplementary Information:**

The online version contains supplementary material available at 10.1007/s00401-020-02257-0.

## Introduction

The giant muscle protein titin (also known as connectin) acts as an essential architectural integrator of striated muscle sarcomeres in heart and skeletal muscle by directing the exact subsarcomeric positions of hundreds of other sarcomeric proteins [14, 28]. Titin is a protein of over 1 µm in length, with a molecular weight in excess of 3 MDa, and is encoded by the *TTN* gene, which assembles the primary ~ 100 kb transcript from 364 exons in a 300 kb gene [2, 8]. The protein contains 132 fibronectin-3 (Fn3) domains, one kinase domain and up to 169 immunoglobulin (Ig) domains, and unstructured regions [29]. In addition to its architectural and mechanical functions in assembling sarcomeres and supporting their contraction and relaxation, the C-terminal, M-band region of titin has emerged as a hub for the coordination of sarcomere proteostasis [33].

Recently, mutations in *TTN* have emerged as a major cause of both dominantly and recessively inherited myopathies covering a wide and still expanding spectrum (for review [8, 53]), including tibial muscular dystrophy (TMD) [21], limb girdle muscular dystrophy 2 J (LGMD2J) [21], hereditary myopathy with early respiratory failure (HMERF) [23, 24, 33, 38, 41, 57], Salih myopathy [4], centronuclear myopathy (CNM) [6], core myopathy with heart disease [7] and childhood-juvenile onset Emery-Dreifuss-like phenotype without cardiomyopathy [10]. There is emerging evidence that many of these presentations form part of a continuum of “congenital titinopathy” [40] rather than distinct entities. *TTN*-related myopathies show considerable clinico-pathological overlap as a group but also with other neuromuscular disorders, in particular the congenital myopathies (CMs), limb girdle muscular dystrophies (LGMDs) and distal myopathies. *TTN* mutations, whether recessive or dominant, are also emerging as major causes of dilated cardiomyopathy [3, 18, 43, 59, 60] and have been implicated in hypertrophic cardiomyopathy (HCM) [35], however no families with co-segregation of *TTN* missense variants with HCM or DCM have been identified to date [1, 56].

An intriguing feature of titinopathies is their often highly restricted disease manifestation, even for missense variants in domains ubiquitously expressed in striated muscle. For example, dominantly-inherited TMD [21] involves almost exclusively the tibialis anterior muscle, despite the *TTN* mutation affecting the ubiquitous interaction between obscurin and titin [12, 44, 45]. Even patients with recessively inherited LGMD2J due to homozygosity for the same *TTN* mutation, despite a more extensive skeletal-muscle phenotype, do not normally develop a cardiomyopathy. Also, mutations in the fibronectin (Fn3)-119 domain, which is expressed in all *TTN* transcripts, are the main cause of HMERF [48, 53]. Cardiac involvement is not a consistent feature of HMERF, although recent studies reveal that some patients may develop conduction abnormalities [55].

To date, around 130 pathogenic *TTN* coding sequence mutations have been reported in association with skeletal and/or cardiac myopathies (for review [8, 53]), the majority truncating loss-of-function mutations (TTNtv). Recessive truncating mutations near the C-terminal M-band have been identified as the cause of Salih myopathy [4], whereas more recently identified recessive mutations in CNM with variable cardiac involvement [6] and other neuromuscular phenotypes also involve the N-terminal region, and comprise compound heterozygosity of either two truncating mutations or a truncation and missense mutation in trans [7, 41]. While there is emerging understanding of the functional consequences of TTNtv, the mechanistic impact of most missense variants and their interplay in compound-heterozygous inheritance with truncating variants is still unclear, resulting in patients with potentially pathogenic *TTN* variants where no definite diagnostic conclusion can be reached due to limitations of pathogenicity ascertainment.

Correct interpretation of *TTN* variants identified in patients presenting with neuromuscular features poses several challenges, mainly reflecting the giant size of the *TTN* gene and the substantial associated spontaneous genetic variation even in healthy individuals. Presence of a *TTN* variant on databases of genetic variation does not necessarily preclude its pathogenicity, as demonstrated for the recessive titin kinase (TK) domain variant Trp260Arg (p.Trp34072Arg using the inferred complete, IC, nomenclature) [7], which, like almost 20 other TK domain variants predicted to be disruptive, is found on the 1000 genomes and other genomic variation databases [17, 34]. Vice versa, absence of a *TTN* variant in large control populations does not necessarily support its pathogenicity, as demonstrated by very rare, often private *TTN* missense variants that are not always predicted to be disruptive [29]. The pathogenicity of TTNtv is supported by their implication in human disease phenotypes such as the primary dilated cardiomyopathies and their relative low frequency on databases of human genetic variation. However, truncating *TTN* variants are carried by about 1:100 of the population [50, 54]. This is much higher than the incidence of DCM by all established causes combined (about 1:500) and also higher than the estimated occurrence of TTNtv of about 1:1000 in end-stage or familial DCM cases [11, 58]. TTNtv therefore may also be carried by entirely healthy individuals. Whilst not without pitfalls for truncating *TTN* variants, pathogenicity assessment is even more challenging for *TTN* missense variants, which account for 90% of *TTN* variants identified in a HCM cohort [35]. Moreover, in contrast to other neuromuscular disorders due to mutations affecting less complex proteins, in silico analysis of *TTN* variants is often severely limited and may even result in predictions conflicting with the results of a more rigorous functional analysis [7]. In addition, clinico-pathological features associated with *TTN* mutations are often insufficiently specific to predict *TTN* involvement confidently, potentially causing further confusion, as the *TTN* variants identified in a specific patient may be coincidental to another Mendelian disorder with overlapping clinico-pathological features. This may be particularly true for recessive missense variants, which are expected to occur in the population at a detectable frequency.

Missense mutations in titin M10 linked to LGMD2J and TMD have previously been biophysically characterised, with mutations deemed causative either preventing correct folding or thermally destabilising the domain by 21 °C, and preventing binding to its ligand Obscurin Ig-1 [52]. Similarly, the impact of HMERF-linked mutations in Fn3-119 have been attributed to misfolding of the domain, based on the inability of soluble mutant protein to be purified from *E. coli* [23, 24].

Due to the now-routine use of next generation sequencing in clinical diagnostics, it is likely that information regarding genetic variation in the *TTN* gene and the group of *TTN*-related myopathies will continue to expand massively in the coming years, emphasizing the need for a structured approach to pathogenicity assessment and to establish reliable genotype–phenotype correlations. Here, we present a comprehensive approach to the assessment of (in particular missense) *TTN* variants in the context of suggestive clinico-pathological features. We use an integrated biochemical and biophysical approach to assess the impact of titin missense variants on protein stability and highlight histopathological and clinical findings more suggestive of a *TTN*–related myopathy compared to other neuromuscular disorders presenting with overlapping features.

## Methods

### Patients

Patients with a clinico-pathological diagnosis of a CM and at least two truncating, splice-site or rare missense variants either in trans or with unknown inheritance in the *TTN* gene were recruited from the participating tertiary neuromuscular centres. Genetic data as well as clinical and pathological features, including those considered to be suggestive of *TTN*-related myopathies (as listed in Oates et al., Tables 1 and 2) [40] were systematically captured. All numbering of genetic variants corresponds to the IC transcript NM_001267550; LRG391_t1.

**Table 1:** Key clinical and histopathological features of patients with *TTN*-related myopathies

Patient	Sex	Age	A/D	Histo	Onset	Function	Contractures	Scoliosis	Surgery	Cardiac	CMP	Resp	Vent	Bulbar
1	M	37	A	CFTD	Neonatal	Sitting	Yes	Yes	No	No	No	Yes	Tracheo	No
2	F	30	A	CNM	Neonatal	Walking	Yes	Yes	ND	Yes	Yes	Yes	NIV	Yes
3	M	12	A	CNM	Infancy	Sitting	Yes	Yes	No	No	No	Yes	NIV	Yes
4	M	10w	D	CNM	Neonatal	NA	No	No	NA	No	ND	Yes	NIV	Yes
5	F	3	A	CNM	Antenatal	Sitting	Yes	Yes	No	Yes	Yes	Yes	Tracheo	Yes
6	M	14	A	MmD	Infancy	Walking	No	Yes	ND	No	No	No	NA	No
7	F	12	A	MmD	Childhood	Walking	No	No	ND	No	No	Yes	NA	No
8	M	8	A	CFTD	Neonatal	Walking	No	Yes	No	No	No	Yes	NIV	Yes
9	F	23	A	CNM	Childhood	Walking	Yes	Yes	No	No	No	Yes	NA	No
10	M	19	A	CNM	Childhood	Walking	Yes	Yes	No	No	No	Yes	NA	No
11	M	14	A	CFTD	Childhood	Walking	ND	Yes	Yes	No	No	No	NA	No
12	M	16	A	CFTD	Antenatal	Walking	Yes	Yes	Yes	No	No	Yes	NIV	No
13	M	11	A	CM	Infancy	Walking	Yes	No	NA	No	No	Yes	NA	No
14	F	24	A	CNM	Neonatal	Walking	Yes	Yes	Yes	Yes	Yes	Yes	NIV	Yes
15	M	21	A	T1P	Antenatal	Walking	Yes	Yes	Yes	Yes	Yes	Yes	NA	Yes
16	F	13	A	ND	Antenatal	Walking	No	Yes	NA	Yes	Yes	Yes	NA	No
17	M	19	A	MmD	Infancy	Walking	Yes	No	No	No	No	Yes	NIV	No
18	M	71	A	MmD	Childhood	Walking	Yes	Yes	No	Yes	Yes	Yes	NA	Yes
19	F	54	D	ND	Infancy	Walking	ND	Yes	No	Yes	Yes	Yes	NA	ND
20	F	50	A	MmD	Neonatal	Walking	No	Yes	No	Yes	Yes	No	NA	No
21	M	47	A	MmD	Neonatal	Walking	No	ND	ND	Yes	Yes	Yes	ND	No
22	M	*18*	*A*	MmD	Neonatal	Walking	Yes	Yes	ND	Yes	Yes**	No	NA	ND
23	M	*3 m*	D	CNM	Antenatal	NA	ND	ND	NA	ND	ND	Yes	NIV	Yes
24	M	9	A	MmD	Neonatal	Walking	No	Yes	Yes	Yes	Yes**	Yes	NIV	No
25	M	31	A	CM	Infancy	Walking*	Yes	Yes	Yes	No	No	Yes	NIV	No
26	F	25	A	CM	Infancy	Walking	No	Yes	No	No	No	Yes	NA	No
27	F	27	A	MmD	Antenatal	Walking (S)	No	ND	ND	Yes	No	Yes	NA	Yes
28	M	10	A	CM	Neonatal	Walking (S)	Yes	Yes	No	Yes	Yes	Yes	NIV	Yes
29	M	32	A	CNM	Neonatal	Walking	Yes	No	NA	Yes	Yes	Yes	NA	No
30	F	32	A	CM	Antenatal	Walking	Yes	Yes	Yes	No	No	Yes	NIV	No

*F* female, *M* male, *A* Alive, *D* Dead, *Histo* Histology, *CNM* Centronuclear Myopathy, *CFTD* Congenital Fibre Type Disproportion, *MmD* Multi-minicore Disease, *CM* congenital myopathy with multiple or non-specific features, *T1P* Type 1 predominance, *Cardiac* cardiac involvement, *CMP* cardiomyopathy, *Resp* respiratory involvement, *Vent* Ventilation, *Bulbar* involvement

*Lost ambulation at 14 years of age

**Underwent cardiac transplant

**Table 2:** Details of the TTN variants identified in our cohort

Patient	*TTN* Mutation 1								*TTN* Mutation 2								Cardio-myopathy	Other *TTN*	Other variants
	Nucleotide	Amino acid	Description	Region	Domain	Expressed in transcript?			Nucleotide	Amino acid	Description	Region	Domain	Expressed in transcript?					
						N2AB	N2A	N2B						N2AB	N2A	N2B			
1	c.470C > A	p.Ala82Asp	Missense	Z-disk	Ig1	Y	Y	Y	c.104646_104650del	p.Arg34807Serfs*7	Frameshift	M-band	Between Ig-162 & Ig-163	Y	Y	Y	N	No	*RYR*
2	c.2140C > T	p.Gln639*	Nonsense	Z-disk	Between Ig-2 and Ig-3	Y	Y	Y	c.83394_83395delGG	p.Glu27723Aspfs*10	Frameshift	A-band	Ig-141	Y	Y	Y	Y	No	
3	c.4949_4953delTGAAA	p.Met1575Serfs*6	Frameshift	Z-disk	Ig-7	Y	Y	Y	c.22186G > A	p.Glu7321Lys*	Splicesite	I-band	Between Ig-57 and Ig-58	Y	Y	N	N	No	*PLEC*
4	c.4949_4953delTGAAA	p.Met1575Serfs*6	Frameshift	Z-disk	Ig-7	Y	Y	Y	c.22186G > A	p.Glu7321Lys*	Splicesite	I-band	Between Ig-57 and Ig-58	Y	Y	N	N	No	*PLEC*
5	c.6206C > A	p.Ser1994*	Nonsense	Z/I Junction	Between Ig-9 and Ig-10	Y	Y	Y	c.105751C > T	p.Gln35176*	Nonsense	M-band	Ig164	Y	Y	Y	Y	No	
6	c.14596 + 1_14597dup	p.?	Duplication	I-band	Ig-31	Y	Y	N	c.16021C > T	p.Arg5266*	Nonsense	I-band	Ig-36	Y	Y	N	N	Yes	
7	c.14596 + 1_14597dup	p.?	Duplication	I-band	Ig-31	Y	Y	N	c.16021C > T	p.Arg5266*	Nonsense	I-band	Ig-36	Y	Y	N	N	Yes	
8	c.15385T > C	p.Cys5054Arg	Missense	I-band	Ig-33	Y	Y	N	c.74020delT	p.Tyr24599Metfs*12	Frameshift	A-band	Fn3-66	Y	Y	Y	N	No	
9	c.19651 + 2 T > A	N/A	Splicesite	I-band	Between Ig-48 & Ig-49	Y	Y	N	c.60352G > T	p.Gly20043*	Nonsense	A-band	Fn3-33	Y	Y	Y	N	Yes	
10	c.19651 + 2 T > A	N/A	Splicesite	I-band	Between Ig-48 & Ig-49	Y	Y	N	c.60352G > T	p.Gly20043*	Nonsense	A-band	Fn3-33	Y	Y	Y	N	Yes	
11	c.24976C > T	p.Gln8251*	Nonsense	I-band	Ig-67	Y	Y	N	c.106756 + 5G > A	N/A	Splicesite	M-band	Between Ig-166 & Ig-167	Y	Y	Y	N	Yes	*NEB*
12	c.25229C > A	p.Ser8335*	Nonsense	I-band	Ig-68	Y	Y	N	c.32920A > T	p.Lys10899*	Nonsense	I-band	PEVK	Y	Y	N	N	No	
13	c.37309delG	p.Ala12362Leufs*584	Frameshift	I-band	Between Ig-86 and Ig-87	N	N	N	c.44506 + 1G > A	N/A	Splicesite	I-band	Between Ig-98 & Ig-99	Y	Y	Y	N	No	
14	c.40783G > C	p.Val13520Leu	Splicesite	I-band	PEVK	Y	Y	Y	c.69646_69647insAAAAG;	p.Gly23141Glufs*37	Frameshift	A-band	Fn3-56	Y	Y	Y	Y	No	
15	c.41369C > A	p.Ala13715Glu	Missense	I-band	Ig-87	Y	Y	Y	c.33289C > T	p.Arg11022*	Nonsense	I-band	PEVK	Y	Y	N	Y	No	
16	c.41369C > A	p.Ala13715Glu	Missense	I-band	Ig-87	Y	Y	Y	c.33289C > T	p.Arg11022*	Nonsense	I-band	PEVK	Y	Y	N	Y	No	
17	c.48624C > A	p.Asn16133Lys	Missense	A-band	Fn3-4	Y	Y	Y	c.16147C > T	p.Arg5308*	Nonsense	I-band	Ig-36	Y	Y	N	N	No	
18	c.49638G > C	p.Trp16471Cys	Missense	A-band	Fn3-7	Y	Y	Y	c.52234C > T	p.Arg17337*	Nonsense	A-band	Ig-112	Y	Y	Y	Y	No	
19	c.49638G > C	p.Trp16471Cys	Missense	A-band	Fn3-7	Y	Y	Y	c.52234C > T	p.Arg17337*	Nonsense	A-band	Ig-112	Y	Y	Y	Y	No	
20	c.51376T > C	p.Cys17051Arg	Missense	A-band	Fn3-12	Y	Y	Y	c.98352_98362delATACCAAGAAG	p.Gln32711Leufs*22	Frameshift	A-band	Ig155	Y	Y	Y	Y	No	
21	c.51376T > C	p.Cys17051Arg	Missense	A-band	Fn3-12	Y	Y	Y	c.98352_98362delATACCAAGAAG	p.Gln32711Leufs*22	Frameshift	A-band	Ig155	Y	Y	Y	Y	No	
22	c.66920T > A	p.Val22232Glu	Missense	A-band	Fn3-49	Y	Y	Y	c.102058delA	p.Asn34020Thrfs*9	Frameshift	A/M Junction	Kinase	Y	Y	Y	Y	No	
23	c.76664G > C	p.Arg25480Pro	Missense	A-band	Fn3-73	Y	Y	Y	c.44260C > T	p.Arg14679*	Nonsense	I-band	Ig-98	Y	Y	Y	ND	No	
24	c.83771G > T	p.Gly27849Val	Missense	A-band	Fn3-90	Y	Y	Y	c.63826C > T	p.Arg21201*	Nonsense	A-band	Ig-123	Y	Y	Y	Y	Yes	
25	c.95420C > T	p.Pro31732Leu	Missense	A-band	Fn3-119	Y	Y	Y	c.95420C > T	p.Pro31732Leu	Missense	A-band	Fn3-119	Y	Y	Y	N	No	
26	c.95420C > T	p.Pro31732Leu	Missense	A-band	Fn3-119	Y	Y	Y	c.95420C > T	p.Pro31732Leu	Missense	A-band	Fn3-119	Y	Y	Y	N	No	
27	c.95765G > C	p.Arg31847Pro	Missense	A-band	Fn3-120	Y	Y	Y	c.16513C > T	p.Arg5430*	Nonsense	I-band	Ig37	Y	Y	N	Y	Yes	
28	c.97013_97014delCT	p.Thr32263Serfs*5	Frameshift	A-band	Fn3-123	Y	Y	Y	c.106756 + 1G > A	N/A	Splicesite	M-band	Between Ig-166 & Ig-167	Y	Y	Y	Y	No	
29	c.97546_97574del	p.Asp32441Phefs*1	Frameshift	A-band	Fn3-123	Y	Y	Y	c.106584G > A	p.Trp35453*	Nonsense	M-band	Ig-166	Y	Y	Y	Y	Yes	*MYBPC-3*
30	c.102439T > C	p.Trp34072Arg	Missense	A/M Junction	Kinase	Y	Y	Y	c.23611C > T	p.Arg7796*	Nonsense	I-band	Ig-63	Y	Y	N	N	No	

### Muscle biopsy

Muscle biopsies were performed as part of the routine diagnostic work-up. Histological, histochemical, immunohistochemical stains and ultrastructural studies were performed according to standard protocols.

### Muscle imaging

Muscle MRI, CT and/or US images mainly from the pelvis and the lower limbs were obtained from selected patients applying standard protocols.

### Genetic studies

Sequencing of the *TTN* coding sequence was performed as part of the normal diagnostic process or through whole exome sequencing as part of a research study. Some details from Patient 22, who presented with the truncating mutation (p.Asn34020Thrfs*9) and Val22232Glu missense variant have been reported previously as Patient 4 in [7].

### Bioinformatics

The impact of missense variants in the study cohort were predicted using various bioinformatic predictors. CONDEL [20], mCSM [49] and Q(SASA) values [5] and gnomAD minor allele frequencies [34] were retrieved from TITINdb [29]. CADD Phred scores for the missense variants in this study, alongside all Titin missense variants in the ClinVar [30] and GnomAD databases were retrieved from the CADD database [51].

### Molecular cloning

For bacterial protein expression, DNA encoding titin domains Ig-1, Ig-33, Ig-87, Fn3-4, Fn3-7, Fn3-12, Fn3-20, Fn3-49, Fn-69, Fn3-73, Fn3-90, Fn3-119 and Fn3-120 were amplified from a human skeletal-muscle cDNA library and inserted into modified pET6His vectors. For domain boundaries see Supplementary Table 1. Missense variants Ala82Asp (Ig-1), Cys5054Arg (Ig-33), Ala13715Glu (Ig-87), Asn16133Lys (Fn3-4), Trp16471Cys (Fn3-7), Cys17051Arg (Fn3-12), Leu18237Pro (Fn3-20), Val22232Glu (Fn3-49), Arg24947Cys (Fn3-69), Arg25480Pro (Fn3-73), Ile27775Val (Fn3-90), Gly27849Val (Fn3-90), Pro31732Leu (Fn3-119) and Arg31847Pro (Fn3-120) were introduced into the wild type vectors by point mutagenesis.

For expression in mammalian cells, DNA encoding two titin regions spanning Ig-125, Fn3-47—Fn3-49, Ig-126 (Ig-125-126) and Ig-141, Fn3-90, Fn3-91, Ig-142 (Ig-141-142) were amplified from a human skeletal-muscle cDNA library and inserted into a pCMV-GFPC2 vector. The Val22232Glu (Fn3-49) and Gly27849Val (Fn3-90) variants were introduced by point mutagenesis.

### Protein expression and purification

pET6His vectors encoding 6-histidine-tagged titin WT and variant domains were transformed into *E. coli* strains (DE3)-BL21 (Agilent) RIPL or K12 Shuffle (NEB, Ig-87 only) and cultured in Luria Bertani or Terrific Broth media at 18 °C overnight following induction of protein expression by addition of isopropyl-b-D-thiogalactopyranoside to 0.5 mM at an absorbance at 600 nm of 0.4–0.8.

For Western blot solubility analysis in bacteria, ~ 1 mL cell pellet was resuspended in BugBuster (Merck) supplemented with DNase I and cOmplete^®^ protease inhibitor (Sigma). Following incubation at room temperature for 30 min, the soluble and insoluble fraction were separated by centrifugation at 18,000 g at 4 °C for 30 min. In HEK293, cells were washed in PBS, lysed in Tris-buffered saline with 0.4% NP-40, 2 mM DTT and the soluble and insoluble fractions were separated as above. Samples corresponding to the total and soluble fractions were mixed with SDS-PAGE sample buffer.

For biophysical analysis, 1 L cell pellets were re-suspended in lysis buffer supplemented with protease inhibitors, incubated with lysozyme and sonicated on ice. Following incubation with DNase I, the soluble and insoluble fractions were separated by centrifugation at 17,000 g for 40 min at 4 °C. Proteins expressed in the soluble fraction were purified by nickel affinity chromatography followed by size exclusion chromatography. Proteins expressed in the insoluble fraction were solubilised in 8 M urea and purified by nickel affinity chromatography under denaturing conditions. Attempts were made to refold the domains by step-wise dialysis into lower concentrations of urea, followed by size exclusion chromatography.

### Western blotting

Total and soluble protein fractions from expression tests were separated by SDS-PAGE on a 4–20% precast gel (BioRad) and low-density agarose-assisted polyacrylamide gels were used for running patient tissue samples, with both then transferred to nitrocellulose. Expressed protein was detected by incubation with anti-HIS (Novagen 70796, 1:1000 dilution) or anti-GFP (Roche 11814460001, 1:1000) primary antibody followed by HRP-tagged anti-mouse secondary antibody (DAKO P0260, 1:1000). Endogenous titin in patient samples was detected by incubation with anti-titin antibodies T12 [13], A170, M2, M8-M9 and Mis6 (generated for this work, Dundee Cell Products, UK) followed by incubation with secondary antibody as above. HRP activity was measured on a BioRad imager following incubation with chemiluminescent substrate (GE Healthcare).

### Differential scanning fluorimetry

The melting temperature (T_m_) was obtained for selected titin domains according to the protocol outlined in Niesen et al. [39]. 20 µM protein was mixed with 20 X SYPRO Orange (ThermoFisher), dispensed in a BioRad qPCR plate, and heated from 25–95 °C at a rate of 1 °C/min in a MX3005p qPCR machine (Agilent). Fluorescent emission at 610 nm following excitation at 492 nm was measured, with the resulting curve defined in Excel, and the T_m_ calculated in GraphPad (Prism).

### Circular dichroism spectroscopy

The UV and CD spectra of selected titin domains at 40 µM were acquired on an Applied Photophysics Chirascan Plus spectrometer at a path length of 0.5 mm (190–260 nm) or 10 mm (240–400 nm). For thermal denaturation experiments, from 20 °C, samples were cooled to 6 °C, heated to 94 °C at a rate of 1 °C/min with a 2 °C step size and then cooled back down to 6 °C.

### Nuclear magnetic resonance

1D 1H spectra of selected titin domains at 100–200 µM were acquired at 500 and 700 MHz on Bruker Avance spectrometers equipped with cryoprobes at 298 K. Water suppression was achieved by Watergate in combination with excitation sculpting [25]. A total of 8192 points were recorded for each FID with a spectral width of 16 ppm. Prior to Fourier transformation of the FIDs exponential line broadening with a line width of 8 Hz was used. Spectra were processed using Topspin 3.2 (Bruker).

### Mammalian cell transfection, fixation and immunostaining

NRCs were prepared according to [31], and C2C12 and HEK293 cells were prepared following standard protocols. NRCs, C2C12 and HEK293 cells were transfected using Escort III (Sigma), Lipofectamine 3000 (Invitrogen) and Escort IV (Sigma), respectively, according to manufacturer’s instructions. Following incubation at 37 °C, 5% CO_2_ for 24–48 h for NRCs and HEK293 cells and up to 14 days for C2C12 myoblasts to allow differentiation into myotubes, cells were prepared according to [12]. Cells were stained with primary antibodies against myosin heavy chain (clone A4.1025) [9], obscurin domain O59 [61], titin Z1Z2 [15], p62/SQSTM1 (Abcam, ab41116161) and ubiquitin (Merck, FK2, 04–263) followed by Cy3 or Cy5-conjugated goat anti-rabbit or anti-mouse IgG (H + L) secondary antibodies (Jackson ImmunoResearch ML 115–165-146, 115–175-146, 111–175-144 and BioRad STAR36D549GA) and imaged on an LSM510 (Zeiss) or SP5 (Leica) confocal microscope. Antibodies were diluted 1:100 prior to incubation.

### Quantification of sarcomeric GFP-titin localisation

Line scans of 5–6 sarcomeres in an NRC were averaged to a single sarcomere using the anti-obscurin signal to measure sarcomere length, and the Z-disk/I-band region was defined as the region below the midway positions between the peak of the anti-myosin signal in the A-band and the trough of that signal at the Z-disk. The GFP-titin fragment localisation in the I-band/Z-disk region was quantified as a percentage of total GFP signal in 4–5 different cells per titin fragment.

### Statistics

The statistical significance for differences of CADD Phred scores between the variants in the study, ClinVar and GnomAD cohorts was calculated using the Kruskal–Wallis test, and the differences between WT and variant titin fragment Z-disk localisation in NRCs and their solubility when expressed in HEK293 cells was calculated using the student’s t test.

### Study approval

All individuals were enrolled under appropriate procedures and in accordance with ethical guidelines at their local institutions, with written informed consent obtained from all subjects or their legal guardians for genetic studies. The study was conducted in accordance with approvals by the National Research Ethics Service (NRES) London- West London & GTAC (Reference: 06/Q0406/33), the research ethics committee of Great Ormond Street Hospital (Reference: 00/5802), and Guy’s & St Thomas NHS Foundation Trust (Reference: RJ110/N231).

## Results

### Patient demographics and family histories

Thirty patients from 23 families were included in the study, 19 of them male and 11 female. Twenty-seven patients were still alive and 3 had died, at 10 weeks, 3 months and 54 years of age, respectively. Age at last follow-up (or death) ranged from 10 to 71 years (median 19 years). In 14 patients there was a family history of neuromuscular disorders, and in 11 patients there was a family history of cardiac disease in a first degree relative, comprising both confirmed cardiomyopathies and/or sudden unexplained deaths of presumably cardiac causes. No parent had a skeletal myopathy (Table 1, Supplementary Table 2).

### Clinical features

**Fig. 1:**
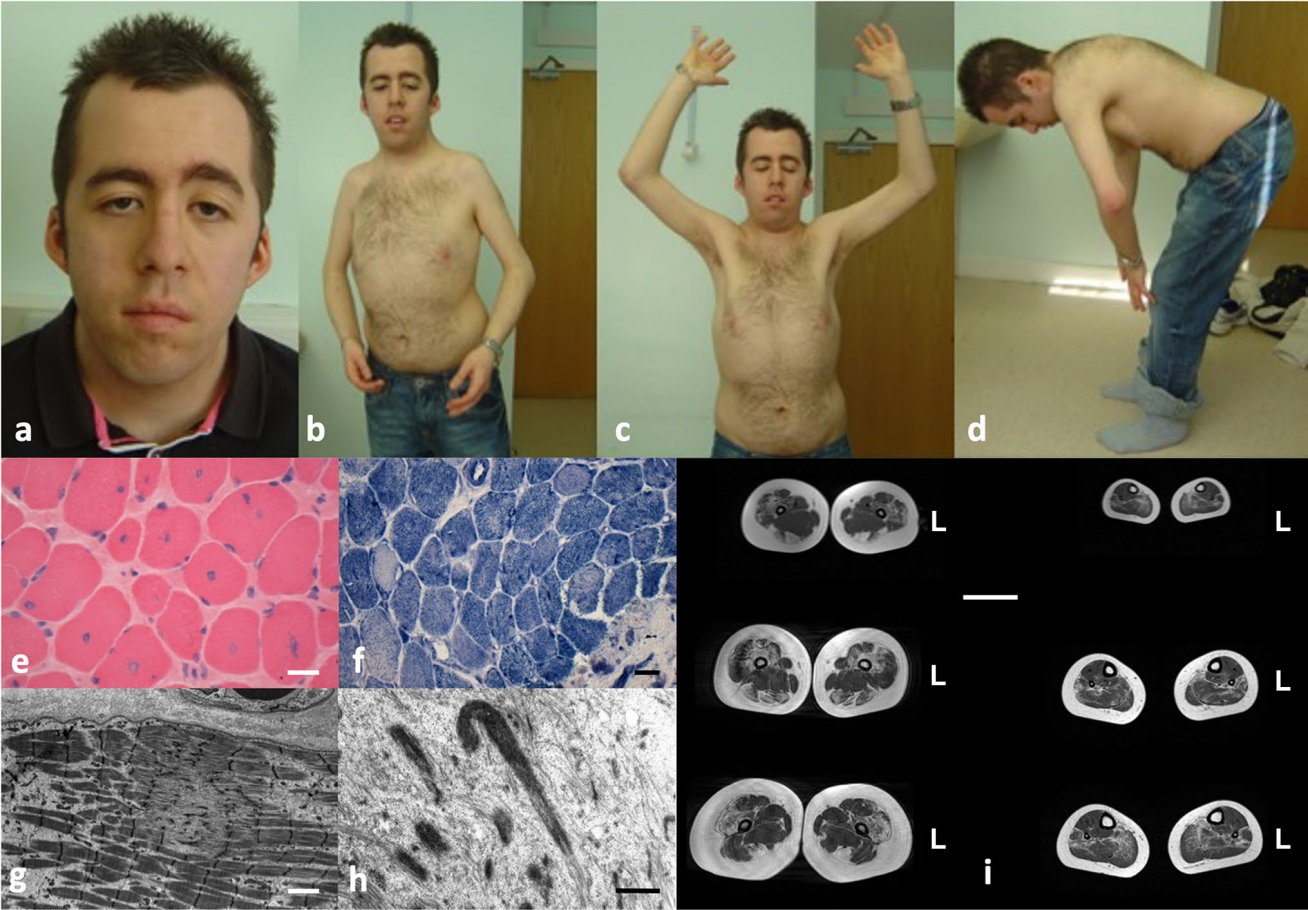
Typical features in *TTN*-related myopathies. This 38-year-old patient presented with hypotonia and ventricular non-compaction from birth. Subsequently, his motor development was delayed. His stature was short, he had a myopathic face with pronounced ptosis (**a**), multiple contractures prominently involving the elbows and shoulders (**b**, **c**), and spinal rigidity (**d**). He developed a dilated cardiomyopathy from his teens. The most prominent feature on muscle biopsy are numerous centralized nuclei (**e**) leading to an initial diagnosis of CNM, but there were additional cores (**f**, **g**) and few nemaline rods (**h**) on EM. Scale bars 40 µm (**e**, **f**), 2 µm (**g**) and 1 µm (**h**). Lower extremity muscle MRI from another patient showing prominent hamstring involvement in the thigh (**i**). Scale bar 5 cm, L indicates left side

The key clinical features are summarized in Table 1 and illustrated in Fig. 1. Presentation was most commonly in the neonatal period (*n* = 11) or antenatally (*n* = 7), and less frequently later in infancy (*n* = 7) or early childhood (*n* = 5). The most common presentation was hypotonia (*n* = 16) and/or weakness (*n* = 14), often pronounced axially, with associated feeding difficulties (*n* = 14) frequently necessitating nasogastric tube feeding (*n* = 6). In two cases, respiratory impairment was severe enough to necessitate intubation and ventilation in the neonatal period. Eight patients had marked dysmorphic features, in the context of congenital cardiac abnormalities, prompting an initial suspicion of Noonan’s syndrome in 2 cases.

Early motor development was delayed in 22 patients. Twenty-three patients achieved and maintained the ability to walk independently for variable distances, except one patient who lost independent ambulation at the age of 14 years. Fifteen patients were at least intermittent wheelchair users. Twenty-one patients showed an overall stable course, whereas progression was rapid in 7, two of whom died within the first year of life from cardiorespiratory causes (no further data available *n* = 2). Patient 19 died aged 54 years, also due to cardiorespiratory causes.

On examination, weakness was typically pronounced axially and proximally. Six patients had additional distal involvement, involving ankle dorsiflexion and the tibialis anterior. Most patients had generalized muscle wasting, but hypertrophy of the calves was found as a rare feature in one family. With few exceptions (*n* = 2), muscle involvement was symmetrical. Patient 15 described distinct episodes of periodic weakness usually occurring after illness or extreme exercise. Combined with the presence of dysmorphic features and a cardiomyopathy, this lead to an initial consideration of a diagnosis of Andersen-Tawil Syndrome; screening for common periodic paralysis-associated genes was negative. Myopathic facial involvement was variable. Ptosis was present in 5 patients (no data *n* = 5) but extraocular muscle involvement was universally absent. Scoliosis (*n* = 22) with or without spinal rigidity (*n* = 13) was very common and in 2 cases already present early in life. Seven patients underwent spinal fusion but others could be managed conservatively. Contractures were a very prominent feature (*n* = 17) and in 7 cases already present at birth. Joint involvement ranged from isolated tendon Achilles tightness to an “Emery-Dreifuss-like” picture, to extensive arthrogryposis affecting most joints and representing a major cause of disability in individual cases. In contrast, many patients had additional or isolated joint hyperlaxity (*n* = 12), often affecting the shoulders, hands, fingers and knees, the latter associated with recurrent patella subluxations in some individuals. Bone fractures (n = 9) after often relatively minor trauma were also prominent, and in one case already present in the neonatal period.

Cardiac involvement was present in 14 patients, comprising congenital cardiac defects (*n* = 4) including atrial septal defects (ASD), ventricular septal defects (VSD) and left-ventricular non-compaction, or a combination of the above, and/or a (dilated) cardiomyopathy (*n* = 13), and/or the presence of arrhythmias (*n* = 10), either in the context of an already existing cardiomyopathy or preceding the latter. Twenty-six patients had evidence of respiratory involvement indicated by either frequent respiratory infections or overt respiratory impairment, with 14 requiring some kind of ventilatory support, ranging from (mainly nocturnal) non-invasive ventilation (*n* = 12) to almost permanent ventilation through a tracheostomy (*n* = 2). Eleven patients had persistent bulbar involvement with chewing and swallowing difficulties, requiring gastrostomy insertion in more than half (*n* = 6) (Table 1, Supplementary Table 2).

### Investigations

Creatine kinase levels were normal in 19 patients and mildly elevated (up to 500 IU/l) in 3 (no data *n* = 8). Electromyography/nerve conduction studies performed in 14 patients were either normal (*n* = 4) or showed mild myopathic abnormalities (*n* = 10) but never neurogenic changes. Thirteen patients underwent muscle imaging, including muscle ultrasound (*n* = 2), muscle CT (*n* = 2) and muscle MRI (*n* = 9). Muscle imaging showed a variable pattern with some consistencies, notably concerning prominent involvement of the paravertebral muscles, the proximal lower limbs, in particular the hamstring muscles, and the tibialis anterior in the lower leg.

### Histopathological features

The principal histopathological diagnoses are listed in Table 1 and Supplementary Table 2, and illustrated in Fig. 1. Based on the most prominent feature on muscle biopsy, the most common histopathological diagnoses in our cohort were, with descending frequency, Multi-minicore Disease (MmD) (*n* = 10), CNM (*n* = 9), Congenital Fibre Type Disproportion (CFTD) (*n* = 5), CM with non-specific or unusual features (*n* = 3) and Type 1 predominance/uniformity (*n* = 1). Two patients did not undergo a muscle biopsy because the histopathological diagnosis had already been established in a sibling with similar clinical features.

The typical histopathological presentation was with a combination of abnormalities rather than a “pure” histopathological picture; these abnormalities included, with descending frequency, (1) core-like structures (*n* = 21) ranging from unevenness of oxidative stains to minicores and, less frequently, central cores (confirmed on electron micrographs where performed); (2) internalized nuclei, often central (*n* = 18); (3) and increased fibre size variability (*n* = 13), often but not always fulfilling the formal criteria for CFTD.

Of note, despite the multitude of other structural abnormalities, nemaline rods were a very rare feature only seen in one patient. Increases in connective tissue and, less frequently, fat were occasionally observed (*n* = 8). Rare ultrastructural findings not previously associated with *TTN*-related myopathies included (“Mallory-like”) cytoplasmic inclusion bodies (*n* = 2) and fingerprint bodies (*n* = 1).

### Genetics

**Fig. 2:**
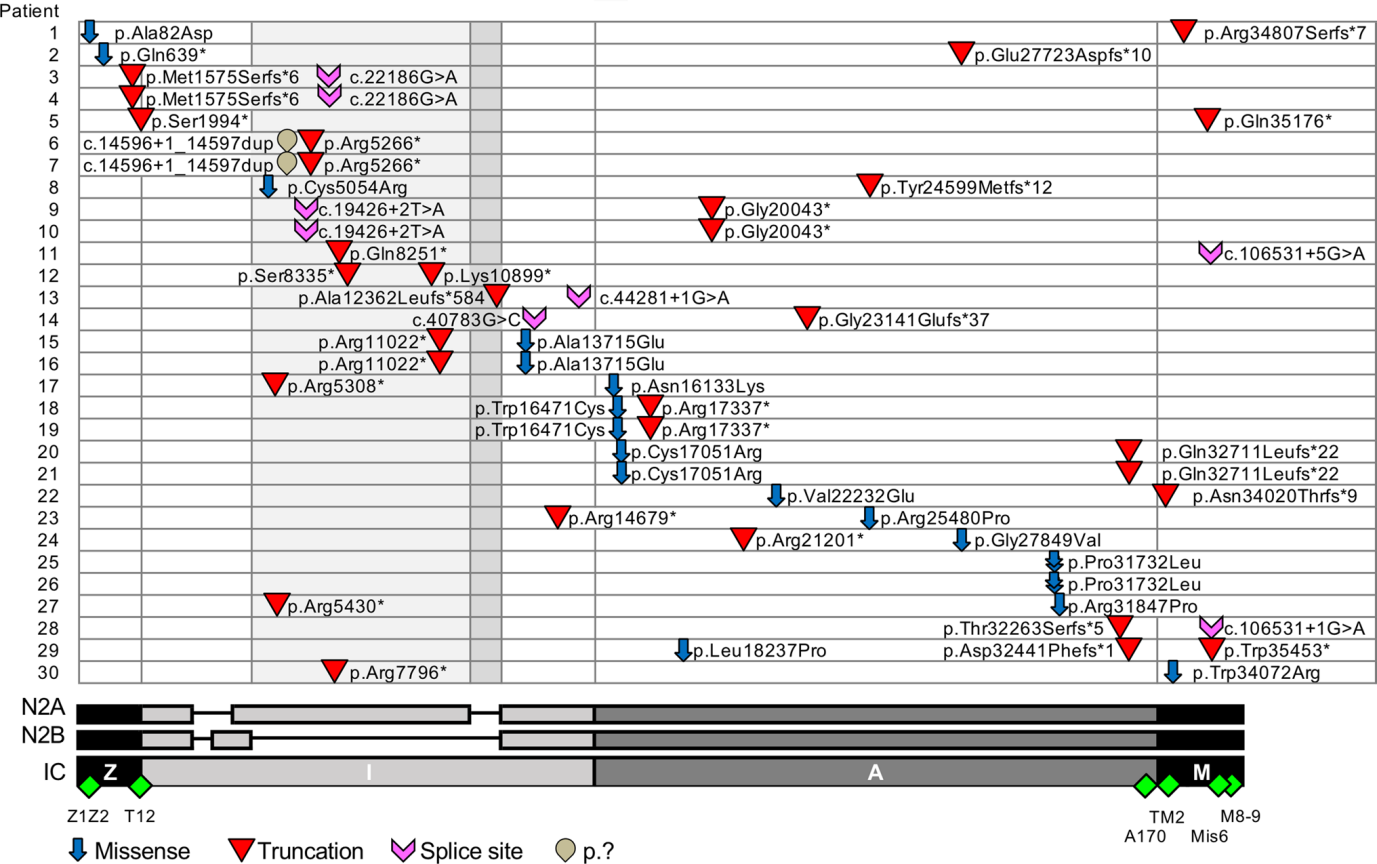
Localisation and type of variants along Titin in our patient cohort. N2A, N2B and IC transcripts detailed, with exons absent in N2B highlighted on plot in light grey and exons absent in both N2A and N2B in dark grey. Positioning of antibodies used in immunofluorescence studies also indicated (green diamonds). DNA and protein numbered according to the IC transcript NM_001267550

*TTN* variants identified in our patients are summarized in Table 2, Supplementary Table 3 and Fig. 2. In 18 patients, compound heterozygosity in trans or homozygosity for *TTN* variants was confirmed; in 3 patients, one *TTN* variant and absence of the second variant was confirmed in one parent; due to lack of any parental DNA, the inheritance pattern of *TTN* variants could not be confirmed in 9 patients. Thirteen of the *TTN* variants identified were missense and 33 variants were expected to be truncating. Three groups of variants were observed: (1) two truncating variants (patients 2, 3, 5–14, 28 and 29), (2) one truncating variant and one missense variant (patients 1, 4, 15–24, 27 and 30) and (3) a homozygous missense variant (patients 25 and 26).

Truncating variants were either stop-gained, insertion/deletion with frameshift or splice donor site variants. Two patients had the same genotype of one truncating variant and two duplications, which were presumed to result in a truncated transcript. For patients with at least one of their two truncating variants occurring in exons whose expression is restricted to skeletal muscle (skeletal tandem-immunoglobulin and PEVK regions), cardiac symptoms were not present at the time of last follow-up.

Eight patients had additional variants in the *TTN* gene: patients 6 and 7 had the second duplication mentioned above. Patient 24 had an additional missense variant in the unfolded PEVK region and patient 27 had one residue deleted in the linker between two immunoglobulin (Ig) domains; both variants are beyond the scope of this study. In addition, patient 24 had a missense variant C-terminal and in cis of a constitutively expressed truncating variant. Patient 29 had a missense variant (Leu18237Pro) in Fn3-20, which is included in this study, in addition to two truncating variants. Finally, patients 9 and 10 had a Leu-to-Val variant in Fn3-27 and patient 11 had an Ala-to-Val variant in Ig-114, respectively. These two variants are currently under investigation, although the conserved nature of the mutations suggests they are non-disrupting and the patient phenotypes can be explained by their two other variants. In 5 patients additional variants were identified in other genes through next generation sequencing, including a potentially pathogenic variant in *MYBPC3* in patient 29 [36] and a variant in *RYR1* previously associated with the malignant hyperthermia (MH) susceptibility trait in patient 1.

Both missense and truncating variants were spread across the entire length of titin and occurred both in differentially spliced regions, notably the I-band region, and constitutively expressed regions, e.g. the Z-disk, A-band and M-band regions (Fig. 2). Except for three *TTN* mutations (Val22232Glu, Pro31732Leu and Trp34072Arg), the missense variants included in the study had not been previously associated with early-onset titinopathies and 8 out of 12 were unique, i.e. not present in gnomAD. The Fn3-119 Pro31732Leu variant (homozygous in patients 25 and 26) has been previously reported as a mutation linked to HMERF, albeit with variable penetrance and considered dominant, recessive, or neutral [32, 41, 46, 47]. The rare *TTN* variant Trp34072Arg (minor allele frequency, MAF 8.21E-06 in gnomAD) in TK (patient 30) has previously been associated with congenital hereditary myopathy (patient 5 in [7]).

### Genotype–phenotype correlations

Several individuals with compound-heterozygous truncating variants were identified with strongly overlapping phenotypes. Compound heterozygous truncating variants were identified predominantly in the I-band and A-band; their pathogenic role in congenital myopathies is increasingly documented [6]. Phenotypic penetrance of truncating variants seems to be entirely driven by exon usage: skeletal muscle-only penetrance is observed when one or both truncating variants reside in an exon not expressed in the cardiac-specific N2B transcript. Cardiomyopathy is not a regular clinical feature in these patients and does not seem prevalent in the parental carriers (Table 2).

Phenotypic penetrance of patients with one truncating and one missense variant is less clear. Whilst four patients with both variants expressed in the predominant skeletal (N2A) and cardiac isoforms (N2B) have been diagnosed with a cardiomyopathy (patients 18, 19, 20 and 24 with an age range of 32–71 years), patient 1 (37-years-old) has not. Additionally, the patients homozygous for the HMERF-linked Pro31732Leu variant in Fn3-119 (patients 25 and 26, 31 and 25-years-old respectively) have not been diagnosed with a cardiomyopathy but show myopathic symptoms only. It should be noted, however, that cardiomyopathy is not a consistent feature for dominantly-inherited HMERF mutations in the Fn3-119 domain, which is expressed in all titin transcripts [55]. The parental carriers and two heterozygous siblings of patients 25 & 26 are unaffected and there is no family history of myopathy or early respiratory failure. Two patients (15 and 16) with the missense variant but not the truncating variant expressed in the N2B isoform of titin have been diagnosed with a cardiomyopathy (Table 2).

### Tissue staining of patients 22, 24 and 29

**Fig. 3:**
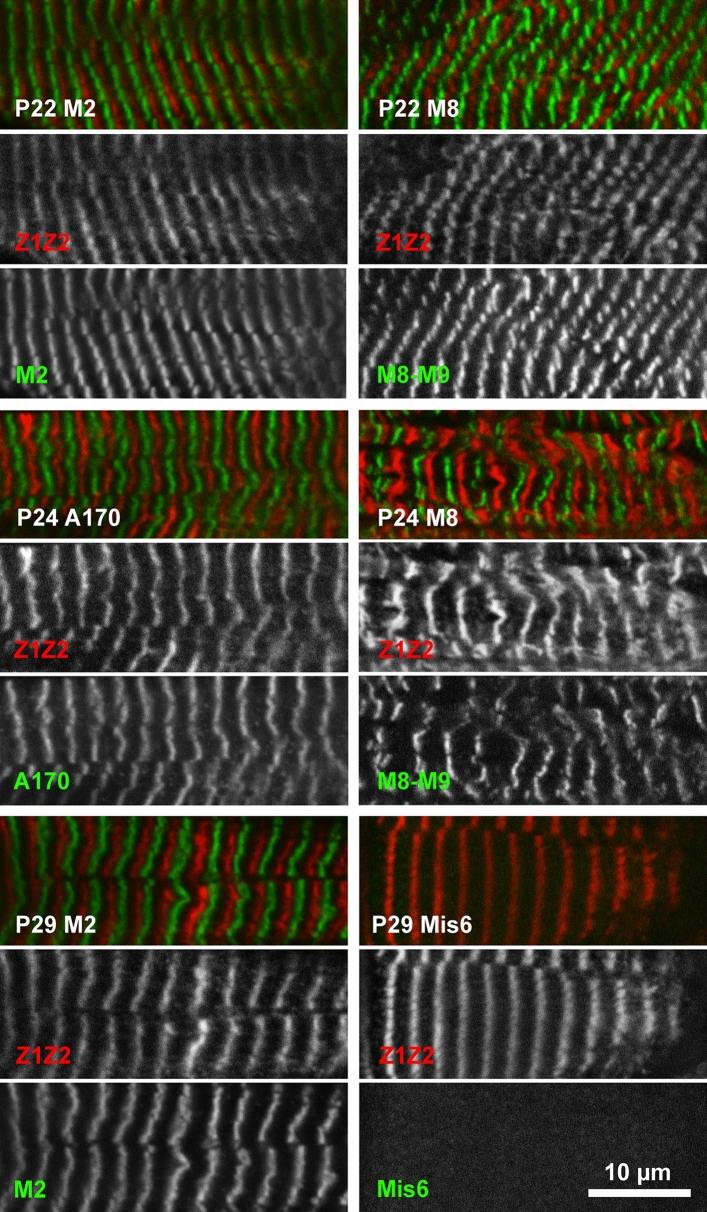
Immunofluorescence analysis demonstrates the sarcomeric integration of truncating and missense titin variants in patients 22, 24 and 29. Cryosections of heart tissue (patients 22 and 24) and skeletal muscle (patient 29) were stained for N-terminal Z-disk titin (red; Z1Z2 antibody) and C-terminal M-band titin (green; M2, Mis6 and M8-M9 antibodies). Note that only the C-terminal epitope Mis6 is absent for patient 29, carrying two truncating mutations before the Mis6 epitope. Scale bar: 10 µm

Heart tissue from patients 22 (truncating variant in the kinase domain and Val22232Glu in Fn3-49), and 24 (p.Gly27849Val in Fn3-90 and p.Arg21201* in Ig-123), and skeletal muscle from patient 29 (truncating variants in Fn3-123 and Ig-166, with an additional Leu18237Pro missense variant in Fn3-20) was available for analysis. All studied truncations are C-terminal to Z-disk titin, which was therefore used as a positional marker for sarcomere integrity (antibodies T12 and Z1Z2) [13, 15] and counter-stained with antibodies against M-band Ig-domains 161 (M2), 167–168 (M8-9) and the inter-domain insertion between Ig-166 and Ig-167 (Mis6 [16]) (Fig. 3). For patients 22 and 24, staining of M-band titin showed that at least one allele was fully translated and integrated into the sarcomere, with no evidence of the missense variant on the non-truncated allele preventing this. For patient 29, staining revealed that titin was integrated into the sarcomere with the Mis6 insertion just N-terminal to the truncation in Ig-166 present, but the most C-terminal staining by an antibody against Ig-167 (M8-9) absent, as predicted by the patient genotype. There was no evidence of exon skipping enabling detectable translation of C-terminal titin (Fig. 3).

### Western blotting of tissue from patients 22, 24 and 29

Western blots of low-density agarose-assisted polyacrylamide gels were run using the muscle tissue described above. Probing the blots with antibodies against epitopes near the N- and C-termini of titin confirmed that the full-length protein was detectable in tissue from patients 22 and 24, but absent in patient 29 (Supplementary Fig. 1), in which the C-terminal epitope Mis6 was not detectable, in excellent agreement with the genotype. A band detected only by antibody T12 was present in patient 24 tissue, as would be predicted for an N-terminal titin fragment of ~ 1.2 MDa, providing evidence of expression of the truncated allele in patient 24.

### Characterisation of missense variants (i–iii)

#### (i) Structural and bioinformatics analyses

**Fig. 4:**
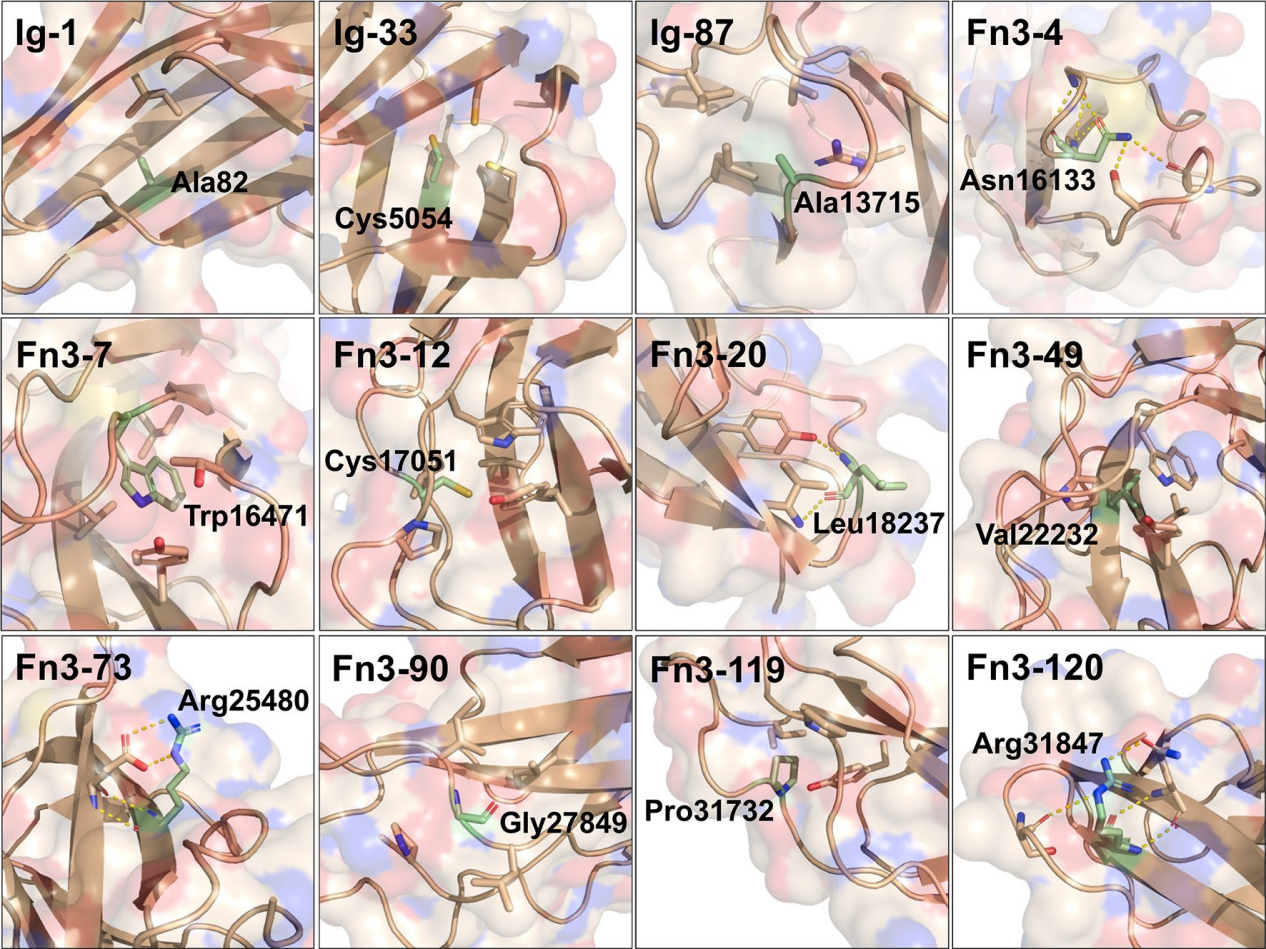
Crystal structures or homology models highlighting atomic environment of residue mutated in patient missense variants. The side-chains of residues (and main chain for Fn3-90) mutated in patients are represented as labelled green sticks, with surrounding side-chains of interest also represented as sticks. Polar contacts are shown via yellow dashed lines. Ig-1 structure is from the crystal structure of first two titin domains, PDB code 2a38. All other models were generated from TitinDB. Images generated using PyMol 2.1.1

Compared to truncating variants, the missense variants pose a significant diagnostic challenge. The 13 missense variants in our cohort that are not predicted to alter splice sites are found in the Ig, Fn3 or kinase domains of titin. The 12 missense variants in the Ig and Fn3 domains were modelled to assess their impact using either crystallographic structures or homology-based structural models generated by TITINdb [29] (Fig. 4). All mutated residues excluding Leu18237 in Fn3-20 are predicted to be in the core of their domain with quotient solvent accessible surface areas, Q(SASA), below 0.15 (residues with Q(SASA) > 0.3 are defined as being solvent accessible) [5] (Supplementary Table 4).

The crystal structure of Ig-1, which harbours the Ala82Asp variant, shows the Ala82 sidechain facing towards the core of the domain, mediating hydrophobic contacts with a neighbouring isoleucine. Mutation to aspartic acid would introduce a charged residue into the core of the domain as well as result in steric clashes with neighbouring residues.

Cys5054 in domain Ig-33 has been implicated in intramolecular disulfide bond formation [37], which have been shown to stabilise immunoglobulin domains [19, 37]. Mutation to arginine would disrupt this disulfide bond formation, with the bulky, charged variant residue likely to further disrupt domain folding. A cysteine-to-arginine variant is also found in Fn3-12 (Cys17051Arg), with this cysteine found at the position typically occupied by the second proline in the PXXP motif at the N-terminal of fibronectin domains. The introduction of a bulky, charged arginine facing towards the core of the domain is likely to disrupt its folding.

Two variants are predicted to mutate residues contributing to the hydrophobic domain core: Trp16471Cys exchanges a canonical tryptophan in Fn3-7 for the small cysteine and Val22232Glu introduces a charged glutamic acid into the core of Fn3-49.

Three variants introduce a bulky sidechain into a loop connecting beta strands, facing inwards towards the domain core: Ala13715Glu in Ig-87, Gly27849Val in Fn3-90 and the HMERF-linked Pro31732Leu in Fn3-119. Asn16133 is similarly positioned in Fn3-4, but the mutation to lysine is predicted to disrupt hydrogen bonding to three neighbouring residues.

Two variants identified mutate an outward-facing beta-sandwich arginine to a proline: Arg25480Pro in Fn3-73 is predicted to abolish a salt bridge with a neighbouring glutamic acid, while Arg31847Pro in Fn3-120 would disrupt hydrogen bonds to two neighbouring residues. In both cases, the main-chain hydrogen bond stabilising the beta sheet contributed by the lysine amide group would be lost.

The missense variant in Fn3-20 found in addition to the two truncating variants in patient 29 is predicted to abolish the main-chain hydrogen bond of Leu18237 to a neighbouring tyrosine side-chain upon mutation to proline. The Trp34072Arg mutation in the kinase domain has been described previously [7].

Strikingly, five of the missense variants mirror mutations in other titin domains linked to disease; that is, the variants structurally align with disease-causing mutations in homology models or crystal structures of other titin domains. Ala82Asp in Ig-1 is the exact structural image of a recently reported Ala178Asp mutation in Ig-2, which was implicated in causing an autosomal dominant cardiomyopathy with features of left-ventricular non-compaction [22]. Four other variants mirror mutations in Fn3-119 linked to HMERF: the Cys17051Arg variant in Fn3-12 mirrors the most frequent HMERF mutation (Cys31712Arg), while Fn3-7 Trp16471Cys, Fn3-4 Asn16133Lys and Fn3-90 Gly27849Val mirror the recently identified HMERF-linked mutations Trp31729Cys, Asn31786Lys and Gly31791Val in Fn3-119, respectively [42]. Additionally, three of these positions in Fn3-119 were also mutated to other residues in the HMERF cohort: Cys31712Tyr, Trp31729Leu, Trp31729Arg, Gly31791Arg and Gly31791Asp.

The missense variants were also assessed using TITINdb for their occurrence in reference SNP databases (gnomAD) and by sequence (Condel) and structure-based (mCSM) modelling of mutational impact (Supplementary Table 4). Condel integrates the output of several sequence-based predictors of mutational impact to provide a “deleteriousness score” between 0 and 1, with variants scoring above 0.5 considered deleterious [20]. The impact of a variant can also be correlated with the changes in the atomic distances surrounding the mutant residue, provided that reliable structural information is available, using the “mutation Cutoff Scanning Matrix” (mCSM) algorithm that predicts changes of free energy (in kcal/mol; [49]). We observed that all missense mutations in the study show negative mCSM values, from − 0.15 to − 2.82 kcal/mol, indicative of a destabilising effect. However, negative mCSM values are also observed for some missense variants in gnomAD with MAF well over 1%. Condel scores all missense variants bar Cys17051Arg and Leu18237Pro as deleterious, but also scores some common missense variants likewise (Supplementary Table 4). This highlights the inadequacy of most commonly used prediction algorithms to reliably assess *TTN* missense variants.

Further bioinformatic analysis was undertaken by comparing the CADD phred scores for the study cohort to scores for all Titin missense variants in the gnomAD and ClinVar cohorts. CADD values are derived from multiple annotations and contrasts simulated variants with those that have survived natural selection, with a higher score for more "deleterious" variants. Thirteen out of the 14 missense variants in the cohort were calculated to be in the top 1% of deleterious missense variants across the genome, but there was no significant difference between the cohort and the Titin gnomAD missense variants (*P* = 0.7). There was a significant difference when comparing the gnomAD and ClinVar cohorts (*P* = 0.04) (Supplementary Fig. 2).

Adding to the ambiguity, the mCSM, Condel and CADD values for each missense variant in the cohort do not correlate (Supplementary Fig. 2). We therefore decided to experimentally validate the stability of wild type and mutant titin domains to ascertain their destabilising effects. Additionally, localisation studies of two missense variants with both cardiac and skeletal phenotypes were performed in cardiomyocytes, skeletal myocytes and a non-muscle model cell line, HEK293 cells.

#### (ii) Expression of wild type and variant domains in bacteria

**Fig. 5:**
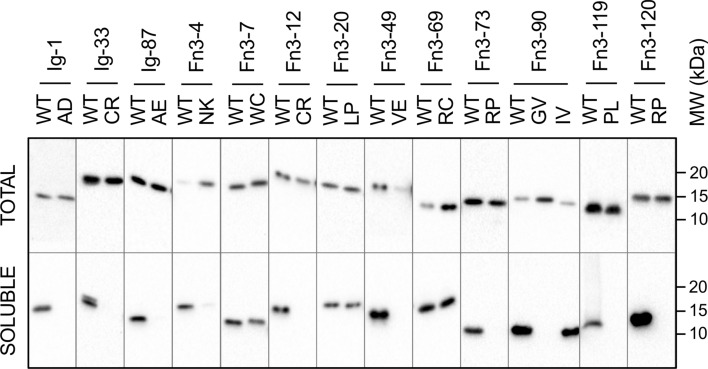
Western blot assessing soluble expression of WT and missense variant-containing titin domains. Bacterial cultures expressing His-tagged titin domains were lysed, separated into total and soluble fractions, run on Western blot and probed with anti-His tag antibody

All titin fibronectin and immunoglobulin domains containing a missense variant in the study cohort were expressed in bacteria, and the expression of wild type and variant recombinant protein was assessed by separating the total and soluble fractions of the cells and assaying via western blot (Fig. 5, Supplementary Figs. 3 and 4). For comparison, we also expressed and assayed two domains with common missense variants (MAF over 0.1) with conflicting classification of pathogenicity: Arg24947Cys in Fn3-69 (classified as deleterious by Condel) and Ile27775Val in Fn3-90 (classified as neutral); their high frequency in the general population rules out a major disease-association. Of the 11 Ig and Fn3 domains tested, only 2 disease cohort variants were solubly expressed (Fn3-7 Trp16471Cys, and Fn3-20 Leu18237Pro), compared to all wild type domains and the common missense variants (Fig. 5; Table 3). This strongly suggested an inability of the bacterial translation machinery to correctly fold the majority of disease-linked missense variant domains.

**Table 3:** Summary of effects of patient cohort and common missense variants on soluble domain expression in *E. coli* and their thermal stability following purification

Patient	Missense variant	Domain	Variant soluble?	WT Tm (°C)	Variant Tm (°C)	Δ domain stability (°C)
1	p.Ala82Asp	Ig-1	No	71.2 ± 0.1 (*n* = 4)	34.0 ± 0.8 (*n* = 4)	− 37.1
8	p.Cys5054Arg	Ig-33	No	–	–	N/A
15 and 16	p.Ala13715Glu	Ig-87	No	–	–	N/A
17	p.Asn16133Lys	Fn3-4	No	–	–	N/A
18 and 19	p.Trp16471Cys	Fn3-7	Yes	75.2 ± 0.6 (*n* = 4)	43.4 ± 0.7 (*n* = 4)	− 31.8
20 and 21	p.Cys17051Arg	Fn3-12	No	–	–	N/A
22	p.Val22232Glu	Fn3-49	No	59.2 ± 0.1 (*n* = 5)	–	Unfolded
23	p.Arg25480Pro	Fn3-73	No	–	–	N/A
24	p.Gly27849Val	Fn3-90	No	61.5 ± 0.2 (*n* = 3)	–	Unfolded
25 and 26	p.Pro31732Leu	Fn3-119	No	49.2 ± 0.1 (*n* = 3)	31.8 ± 0.1 (*n* = 3)	− 17.5
27	p.Arg31847Pro	Fn3-120	No	51.7 ± 0.7 (*n* = 4)/49.7^a^ (*n* = 1)	–	Unfolded
29	p.Leu18237Pro	Fn3-20	Yes	80.4^a^ (*n* = 1)	63.5&78.0^a^ (*n* = 1)	− 16.9/− 2.4
30	p.Trp34072Arg	Kinase	Yes	59^b^ (*n* = 1)	42&57^b^ (*n* = 1)	− 17/− 2
Common variant	p.Arg24947Cys	Fn3-69	Yes	62.6 ± 0.1 (*n* = 3)	62.1 ± 0.2 (*n* = 3)	− 0.5
Common variant	p.Ile27775Val	Fn3-90	Yes	61.5 ± 0.2 (*n* = 3)	63.5 ± 0.1 (*n* = 3)	+ 2.0

Melting temperatures measured using differential scanning fluorimetry (no superscript) or circular dichroism^(a, b)^. Measurements labelled ^(b)^ from (7)

#### (iii) Biophysical characterisation

Five predicted pathogenic domains expressed in the insoluble fraction were purified under denaturing conditions, and attempts were made to refold the protein. Three variants—Val22232Glu (Fn3-49), Gly27849Val (Fn3-90) and Arg31847Pro (Fn3-120)—could not be refolded under any tested condition, as measured by either 1D NMR or circular dichroism (CD).

**Fig. 6:**
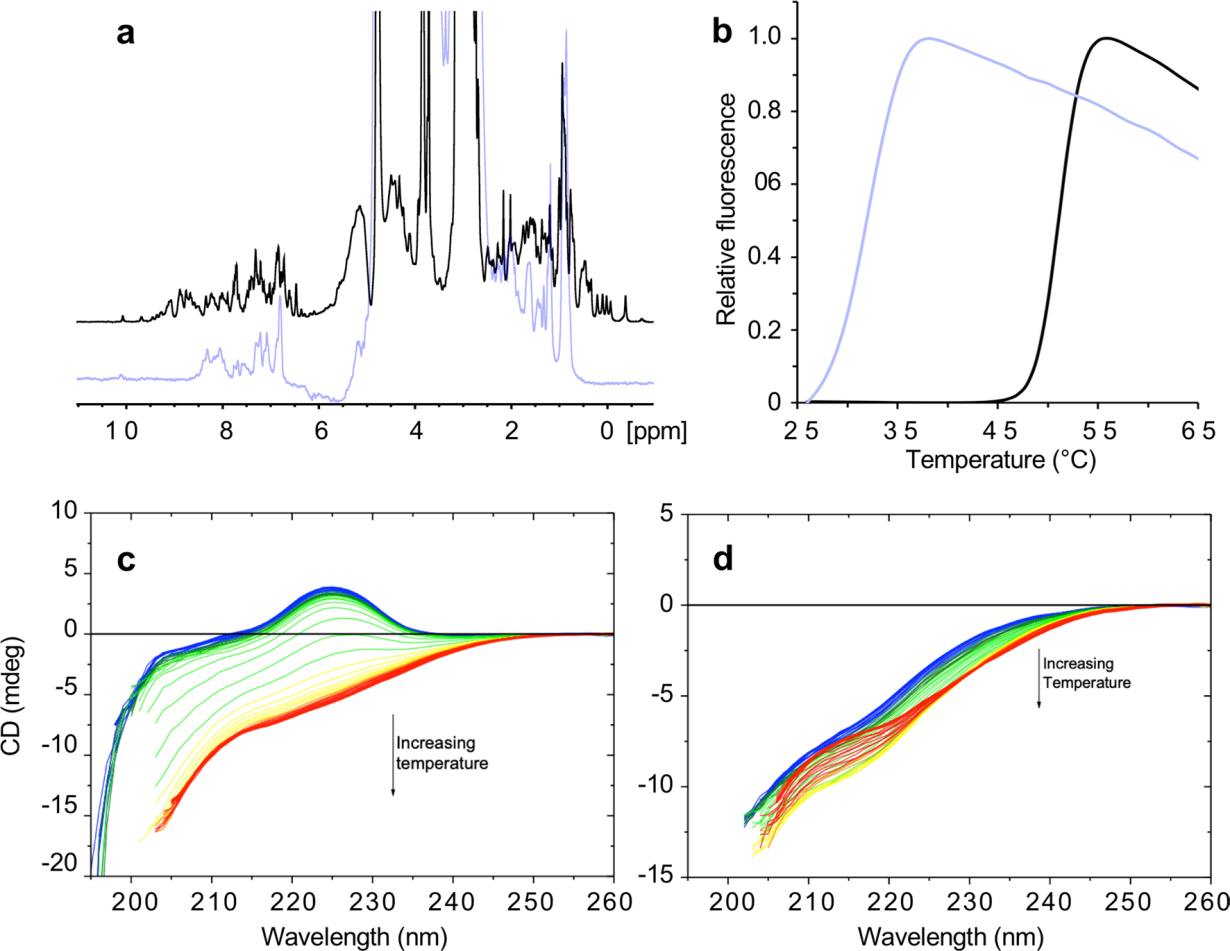
Biophysical characterisation of WT and missense variant-containing titin domains. **a** 1D NMR of Fn3-49 WT (black) and Val22232Glu (lilac). **b** Differential scanning fluorimetry of Fn3-119 WT (black) and Pro31732Leu (lilac). **c** thermal denaturation measured by circular dichroism of Fn3-120 WT (left) and Arg31847Pro (right)

The 1D NMR spectra of Fn3-49 and Fn3-90 WT were typical of a folded protein, with dispersed peaks in the − 1 to 0.5 ppm region corresponding to methyl hydrogens of side-chains in a buried, hydrophobic core and clear dispersion of amide shifts between 6–9 ppm. Conversely, the spectra of Fn3-49 Val22232Glu and Fn3-90 Gly27849Val lacked peaks upfield of 0.5 ppm and presented broader line widths, both indicative of an unfolded protein (Fig. 6, Supplementary Fig. 5).

The conformation and thermal stability of Fn3-20 and Fn3-120 WT and missense domains were assessed by thermal denaturation CD. At 20 °C, the WT domains and Fn3-20 Leu18237Pro had a spectrum typical of a beta sheet-containing protein, with a positive circular dichroism signal around 225 nm, with this signal being lost during heating to 95 °C, due to unfolding of the protein (Fig. 6, Supplementary Fig. 6). Fn3-20 Leu18237Pro showed a two-step unfolding curve, with the first Tm 16.9 °C lower than that the single Tm of the WT domain (Supplementary Fig. 6; Table 3). In contrast to Fn3-120 WT, at 20 °C Fn3-120 Arg31847Pro had no positive CD signal, indicative of an unfolded protein, a state further confirmed by the lack of change in spectra upon heating (Fig. 6). The missense variant Trp34072Arg has previously been shown by this technique to destabilise titin’s kinase domain by 17 °C [7] (Table 3).

The thermal stability of seven domains and their missense variants was also measured using differential scanning fluorimetry (Fig. 6, Supplementary Fig. 7, Table 3). The only disease-linked variant to be solubly expressed, Fn3-7 Trp16471Cys, caused a reduction of the domain’s thermal stability of 31.8 °C. Two variants could be refolded following purification under denaturing conditions: Ig-1 Ala82Asp and Fn3-119 Pro31732Leu. Ala82Asp reduced Ig-1′s thermal stability by 37.1 °C (Supplementary Fig. 7), and Pro31732Leu destabilised Fn3-119 by 17.5 °C (Fig. 6). To benchmark these observations, we assessed the thermal stability of the two common missense variants described earlier. Both variants showed no impact on domain stability in our experimental approach, with Ile27775Val even showing a modest increase in thermal stability (Table 3). As expected for domains that could not be refolded, the variant domains of Fn3-49, Fn3-90 and Fn3-120 did not produce an unfolding curve, in contrast to their corresponding WT domains (Supplementary Fig. 7).

### Localisation of titin fragments in cardiomyocytes

**Fig. 7:**
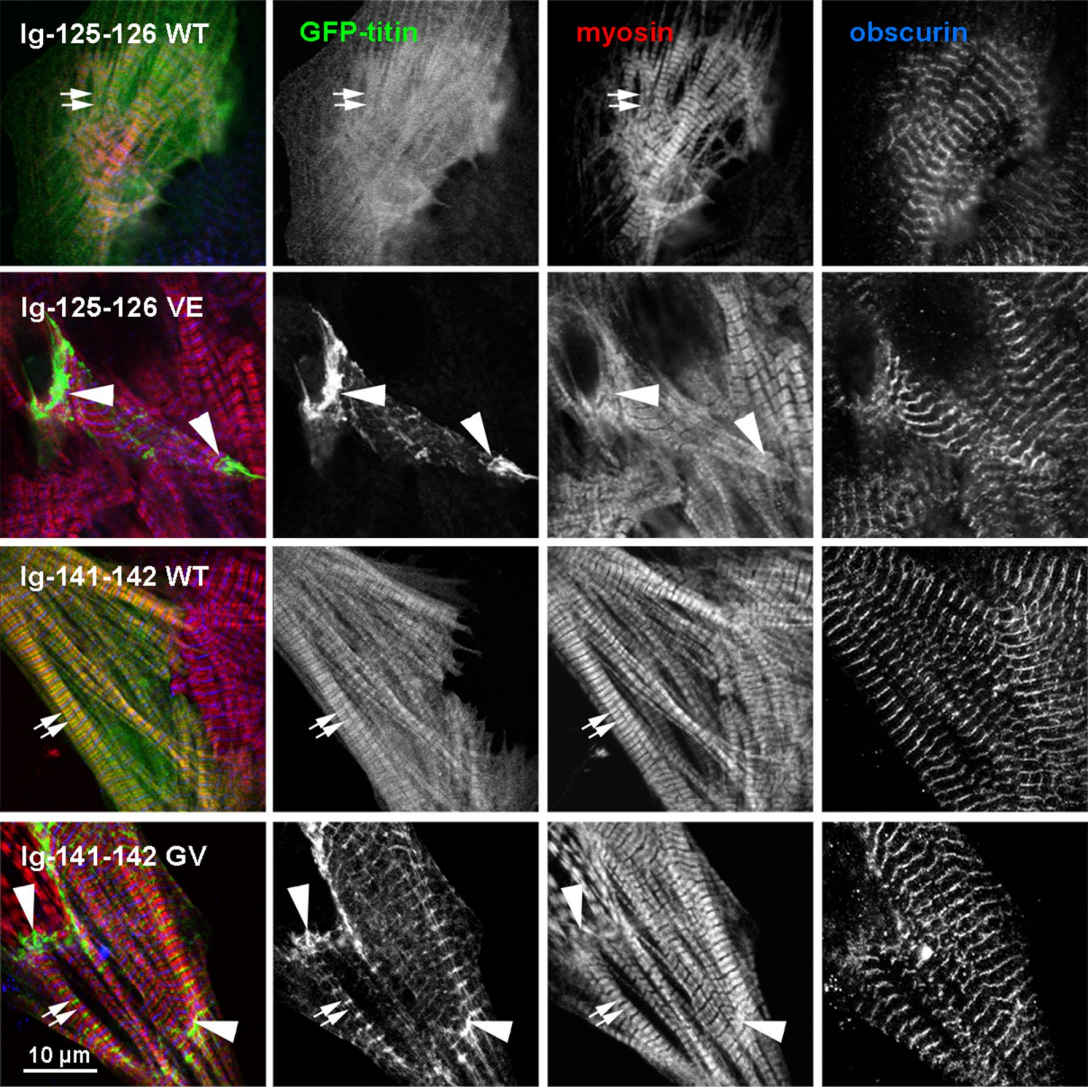
Confocal immunofluorescence microscopy of neonatal rat cardiomyocytes expressing GFP-tagged titin Ig-125-126 WT and Val22232Glu, and Ig-141-142 WT and Gly27849Val. Cells were counterstained with antibodies against myosin heavy chain (red) and Obscurin O59 (blue, marking the M-band); GFP-titin in green. Small arrows mark the A-band doublets labelled by the A4.1025 monoclonal antibody against myosin heads [9] and large arrows mark the intercalated disk. Scale bar: 10 µm

To assess the expression and localisation in a more native environment, WT and variant titin fragments spanning domains Ig-125-126 and Ig141-142, encompassing the Fn3-49 Val22232Glu and Fn3-90 Gly27849Val variants, respectively, were transfected into neonatal rat cardiomyocytes (NRCs). Immunofluorescence confocal microscopy showed a clear difference in the subcellular targeting of the WT and variant constructs (Fig. 7, Supplementary Fig.8). Whilst the expressed WT fragments were diffuse with some localising in doublets with myosin heavy chain (where A-band titin is natively found) the variant fragments showed a significant increase in localisation to the Z-disk (Supplementary Fig. 9). There was also evidence of aggregation, particularly at the intercalated disc, suggesting a failure of the mammalian muscle translation system to correctly fold the variant protein. The functional impact of the misfolded protein therefore appears to extend over several domains, as shown by the mis-localisation of the variant fragments.

### Expression of titin fragments in C2C12 myoblasts

**Fig. 8:**
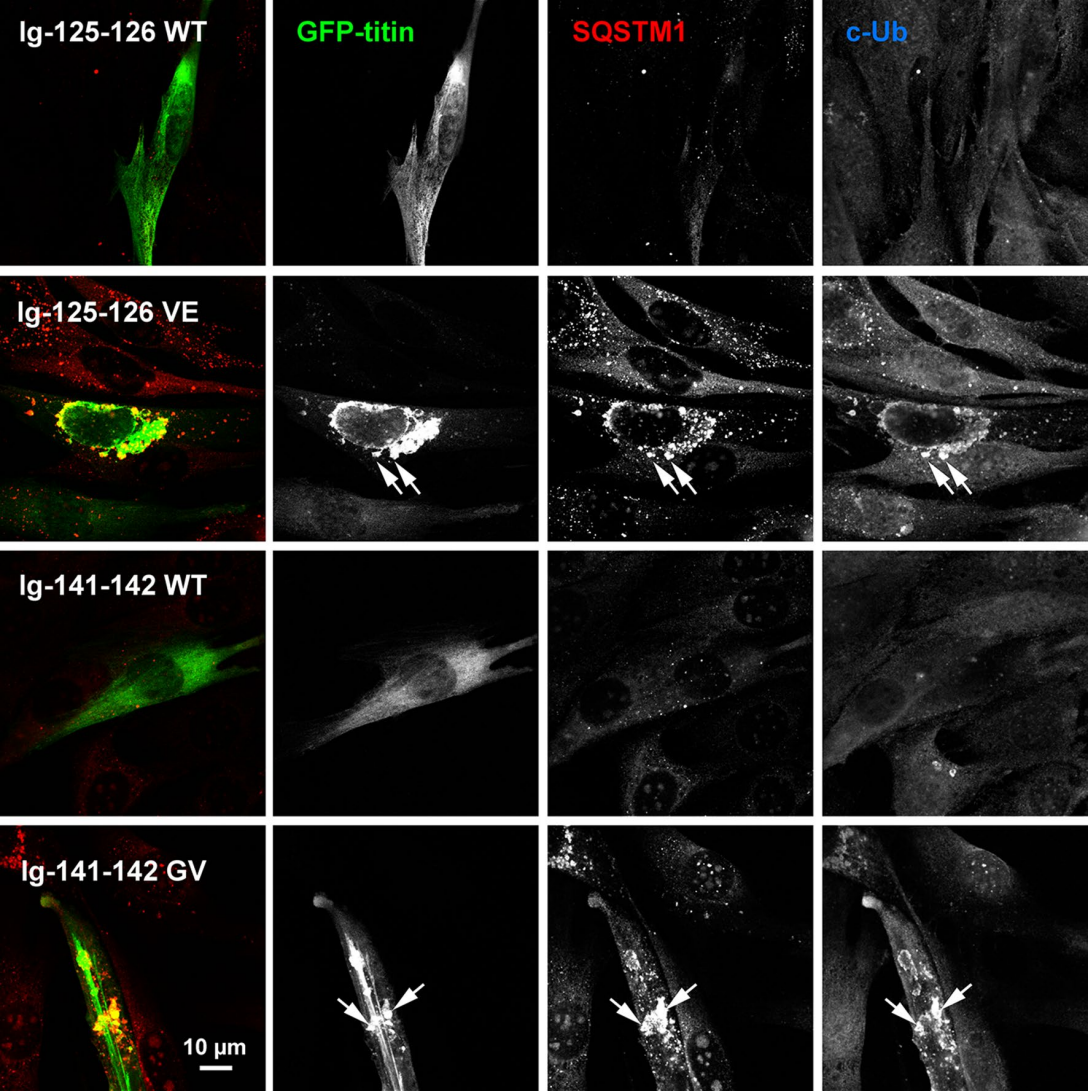
Confocal microscopic images showing expression of titin fragments in C2C12 myocytes. GFP-tagged titin fragments Ig-125-126 WT and Val22232Glu, and Ig-141-142 WT and Gly27849Val, were transfected into C2C12 myoblasts and seven days post-transfection were fixed and stained with antibodies against p62/SQSTM1 and conjugated ubiquitin (c-Ub). 4–8 transfected cells per titin fragment were imaged, with representative cells shown here. Only the GFP and p62/SQSTM1 channels were used for the merged image. Arrows indicate examples of colocalisation of mutant titin, p62/SQSTM1 and conjugated ubiquitin

Ig-125-126 WT and Val22232Glu, and Ig141-142 WT and Gly27849Val were also transfected into C2C12 myoblasts. The WT fragments showed diffuse staining but both missense variants largely formed speckles, frequently colocalising with both p62/SQSTM1, an autophagosome marker, and conjugated ubiquitin, which labels misfolded proteins targeted for degradation (Fig. 8). Only C2C12 myoblasts transfected with WT titin fragments could be cultured for long enough to differentiate into myotubes; no C2C12 myoblasts transfected with variant fragments were observed to differentiate into myotubes (data not shown).

### Expression of titin fragments in HEK293 cells

The four titin fragments described above were transfected into HEK293 cells and their expression was assessed. The WT fragments largely showed a diffuse cytosolic localisation, with infrequent observations of the fragments co-localising with p62/SQSTM1 (Supplementary Fig. 10, Supplementary Fig. 11). In contrast, the variant fragments consistently appeared aggregated, with many examples of colocalisation with p62/SQSTM1. To assess the solubility of the expressed fragments, transfected cells were harvested, lysed and the total and soluble fractions were separated. Western blots showed that the Fn3-49 Val22232Glu and Fn3-90 Gly27849Val variants significantly reduced the fragment solubility compared to WT (Supplementary Fig. 12), mirroring the results observed when expressing the WT and variant single domains in bacteria.

## Discussion

Following introduction of next generation sequencing into routine clinical diagnostics, *TTN* variants are increasingly identified in patients presenting with primary skeletal myopathies. As *TTN* variants are not infrequent amongst control populations, information is needed regarding consistent clinico-pathological features that support the diagnosis of a *TTN*-related myopathy, and how to ascertain pathogenicity confidently, particularly with regards to *TTN* missense variants. In the present study focussing on patients presenting within the congenital myopathy (CM) spectrum, we provide further evidence for consistent clinical and pathological features that support a diagnosis of a *TTN*-related myopathy. We also suggest a comprehensive approach to pathogenicity ascertainment of *TTN* genotypes involving missense variants, beyond the conventional tools of in silico analysis, via the biochemical and biophysical characterisation of domains containing missense variants.

On the clinical level, most patients presented with a severe CM, typically antenatally or from the neonatal period, and less frequently from infancy or later in childhood. Despite the early onset and often substantial motor developmental delay, and in contrast to some other severe CMs (for example, X-linked myotubular myopathy, XLMTM), more than two thirds of our patients achieved and maintained at least some degree of independent ambulation, a remarkable observation also considering the unusually long duration of follow-up in our cohort, with the oldest surviving patient currently 71-years-old. Despite the distinct genotypes, the patients compound heterozygous for one truncating and one missense *TTN* mutation have a very similar phenotype to those with congenital titinopathies compound heterozygous for *TTN* truncating mutations [40]. Multiple contractures, spinal rigidity and scoliosis developed early in the disease course and were frequently progressive throughout life, resulting in clinical phenotypes resembling the King-Denborough syndrome (KDS) or Emery-Dreifuss muscular dystrophy (EDMD). Although the latter phenotype has been suggested as a distinct presentation of *TTN*-related myopathies [10], in our experience there is a continuum of such features throughout the group of patients with *TTN*-related myopathies. Another notable finding in our cohort was the high incidence of fractures that appeared out of proportion to the frequency of this feature in other CM, suggesting a more specific association. In contrast to all other major forms of CNM, extraocular muscle involvement was universally absent. Respiratory involvement was variable but necessitated constant respiratory support in around half of all patients. Cardiac involvement comprised congenital structural cardiac defects, acquired cardiomyopathies and arrhythmias, the latter occasionally preceding the development of more definite cardiac dysfunction. Of note, careful review of the family histories revealed not only unequivocally documented cardiomyopathies but also many unexplained cases of unexpected sudden cardiac death or heart problems attributed to lifestyle factors such as alcohol abuse, which considering our findings may have had a genetic contribution.

On the histopathological level, our findings consolidate *TTN* as a major cause of MmD [7] and CNM [6], but also emphasize that *TTN*-related CMs are most typically associated with a mixed rather than a “pure” histopathological picture, as noted in earlier studies [6, 7]. In contrast to other causes of CNM, in particular XLMTM due to mutations in *MTM1* and *DNM2*, necklace fibres and radial strands were not typically observed.

Taken together, our findings suggest that a *TTN*-related myopathy should be strongly suspected in patients with clinical features of CM with variable cardiac but *without* extraocular muscle involvement, and a mixed histopathological picture with cores and internalized nuclei as the most prominent feature.

Muscle imaging studies suggested a relatively consistent pattern of selective muscle involvement, with considerable overlap with that previously reported in other *TTN*-related myopathies such as TMD and HMERF. However, the pattern of selective muscle involvement did not show the consistency seen in some other neuromuscular disorders (for example, *RYR1*-related myopathies [26, 27]), and larger series will be required to confirm its specificity.

Depending on the location of the truncating *TTN* variants in differentially spliced exons, we are able to propose tentative genotype–phenotype correlations in particular with regards to the degree of cardiac involvement, which should help to inform counselling and health surveillance in individual patients and their families.

Staining and Western blotting of patient tissue showed that C-terminal, M-band titin was present when only one allele harboured a truncating mutation (Fig. 3, Supplementary Fig. 1; patients 4, 22 and 24), suggesting the missense variant-containing allele is incorporated into the sarcomere, but absent when both alleles contained a truncating mutation (patient 29). The staining pattern for patient 29 suggests that at least the longer of the two truncating titin variants is fully integrated into the sarcomere, and there is therefore no evidence of significant nonsense-mediated decay of the truncated alleles. Ordered sarcomeres have been seen previously in patients with two splice site/truncating variants, albeit with disrupted I- and A-band regions [6].

At least eleven of our patients were compound heterozygous for a *TTN* truncating and missense variant, as previously reported in other patients with *TTN*-related CNM [6]. A major emphasis of our study was the detailed functional and biophysical characterization of the *TTN* missense variants identified in our cohort, a particular diagnostic challenge in the ascertainment of *TTN*-related myopathies. Only one previously reported recessive *TTN* missense mutation in CM affecting the TK domain (Trp34072Arg) [7] has been analysed in detail, and was demonstrated to have a strongly destabilizing effect when expressed on a constitutively-truncating background through disruption of the TK scaffolding function for autophagy adaptors Nbr1 and p62/SQSTM1. Although associated with a severe CM with cardiac involvement in the compound-heterozygous state, Trp34072Arg is also present in the general population, as expected for a recessive allele, highlighting an urgent unmet need for appropriate classification of recessive pathogenic *TTN* mutations [8]. It is particularly interesting that this variant was identified again in the unrelated patient 30 in the present study, co-inherited in trans with a truncating variant in a skeletal-muscle specific region. Accordingly, as the compound-heterozygous setting is only expressed in skeletal muscle, the patient shows no cardiac phenotype. We suggest this unequivocally identifies the missense variant as a pathogenic recessive mutation whose phenotypic penetrance is governed by the position of the co-inherited pathogenic *TTN* variant.

We characterized several of the domains mutated in our patients in detail and compared those to similar *TTN* pathogenic variants previously reported (in [8]), demonstrating a similar pattern supporting a disruptive effect of *TTN* missense mutations when expressed on a truncating background. Of note, we characterized the Val22232Glu mutation identified in Patient 4 in [7] (included as patient 22 in our series), affecting Fn3-49 and associated with MmD with additional internalized nuclei presenting from infancy, with a congenital ASD and a VSD, and a rapidly progressive cardiomyopathy requiring cardiac transplantation at the age of 14 years. The variant domain is expressed insolubly in bacteria and remains unfolded following purification from the insoluble fraction. Despite its impact on domain folding, the allele expressing this variant is integrated into the sarcomere, and the variant is recessive with the parental carrier asymptomatic. Similar to Fn3-49 Val22232Glu, 9 of the remaining 11 variant domains were insolubly expressed in bacteria, and Fn3-90 Gly27849Val and Fn3-120 Arg31847Pro could also not be refolded. Ig-1 Ala82Asp and Fn3-119 Pro31732Leu could be refolded, but were highly thermally destabilising, as were the solubly expressed Fn3-7 Trp16471Cys and Fn3-20 Lue18237Pro variants. The mis-localisation of the constructs encompassing Fn3-49 Val22232Glu and Fn3-90 Gly27849Val transfected in cardiomyocytes and their reduction in solubility when expressed in HEK293 cells supports the notion that these mutations convey their pathogenic affects via destabilisation of their domain.

The impact of TMD/LGMD2J-linked mutations in Ig-169 (M10) and HMERF-linked mutations in Fn3-119 have been attributed to misfolding and/or thermal destabilisation of the domains [24]. Alongside Fn3-119 Pro31732Leu, we have also purified Fn3-119 Cys31712Arg (Cys30071Arg in Hedberg et al. [24]), with 1D NMR spectroscopy confirming that these mutants can be refolded following insoluble expression (data not shown), in contrast to the Val22232Glu mutant in CM. The different pathways to disease between variants leading to domains that can or cannot be refolded seems crucial for understanding their pathomechanism and tissue specificity.

It is striking that structural “mirror images” of five of the twelve missense variants in our cohort have previously been linked to titinopathies. Analogous to Fn3-119 being a hotspot for HMERF-linked mutations, particular residue positions within titin domains may be hotspots for titinopathies, at least in trans with truncating variants; this observation may be of importance for the interpretation of missense variants identified in future.

Intriguingly, the clinical phenotype of patients compound-heterozygous for *TTN* truncating/missense variants is very similar to that found in patients carrying two recessive truncating variants, raising the question how these recessive *TTN* variants impact on the titin protein in vivo [6]. Analogous to the TK Trp34072Arg mutation and the HMERF-linked mutations in Fn3-119, the impact of recessive disrupting *TTN* missense variants on proteostasis could be a key pathogenic feature of recessive titinopathies, a hypothesis supported by the observations of variant titin fragments co-localising with p62/SQSTM1 in C2C12 and HEK293 cells. We hypothesise that *TTN* missense variants can derail proteostasis by inducing local cleavage or accelerated protein turnover, causing disintegration of titin (C-terminal fragments created by skeletal-muscle specific calpain-3 in TMD/LGMD2J [10]) or cleavage observed in some CM, or by directly impairing the scaffolding functions of the M-band proteostasis hub [14]. Alterations to cellular proteostasis may be amenable to therapeutic intervention via stabilisation of the domains by chemical chaperones, inductors of endogenous chaperons or by promoting their accelerated degradation.

The present study offers clear strategies addressing the challenges of an accurate assessment of patients with suspected *TTN*-related myopathies, and emphasizes the need for a combined clinico-pathological, genetic and molecular approach to optimize diagnostic accuracy. Rare missense variants should be experimentally assessed at the protein domain level, with thermal destabilisation by more than approximately 15 degrees or complete misfolding/unfolding of the expressed domain supporting a pathogenic diagnosis for the variant when inherited *in trans* with a second *TTN* truncating or destabilising missense variant. These criteria should also prove helpful to assess the possible impact of suspicious missense variants in cardiomyopathies.

## Supplementary Information

Below is the link to the electronic supplementary material.

Supplementary file1 (XLSX 19 KB)

Supplementary file2 (PDF 20076 KB)

Supplementary file3 (XLSX 82 KB)
