# Supplementary material for: Making sense of missense variants in TTN-related congenital myopathies
Source: Acta Neuropathol. 2021 Jan 15;141(3):431–53. doi: 10.1007/s00401-020-02257-0 (PMC7882473; doi:10.1007/s00401-020-02257-0)
Supplement: Supplementary file 2 — Supplementary file2 (PDF 20076 KB) [file 401_2020_2257_MOESM2_ESM.pdf]

Making sense of missense variants in *TTN*-related congenital myopathies

Martin Rees *et al*

Supplementary Online Material

Supplementary Figure 1: Western blots of low density SDS-PAGE gels probing Titin expression in skeletal or cardiac tissue from three congenital myopathy patients.

Supplementary Figure 2: Bioinformatic analysis of titin missense variants.

Supplementary Figure 3: Western blot assessing total expression of WT and missense variant-containing titin domains.

Supplementary Figure 4: Western blot assessing soluble expression of WT and missense variant-containing titin domains.

Supplementary Figure 5: 1D nuclear magnetic resonance of Fn3-90 WT and Gly27849Val.

Supplementary Figure 6: Thermal denaturation circular dichroism of Fn3-20 WT and Leu18237Pro.

Supplementary Figure 7: Differential scanning fluorimetry thermal denaturation curves of selected WT and missense variant-containing titin domains.

Supplementary Figure 8: Confocal immunofluorescence microscopy of neonatal rat cardiomyocytes expressing GFP-tagged titin Ig-125-126 WT and Val22232Glu, and Ig141-142 WT and Gly27849Val.

Supplementary Figure 9: Quantification of titin fragment localization to the Z-disk / I-band.

Supplementary Figure 10: Confocal microscopic images showing expression of titin fragments in HEK293 cells.

Supplementary Figure 11: Bar chart showing classification of localisation of titin fragments expressed in HEK293 cells.

Supplementary Figure 12: Assessment of solubility of WT and variant titin fragments expressed in HEK293 cells.

Supplementary Table 1: DNA and protein phasing for domains expressed in this study, corresponding to the Titin IC transcript (NM_001267550).

Supplementary Table 4: *In silico* predictions for the effect of patient and common missense mutations.

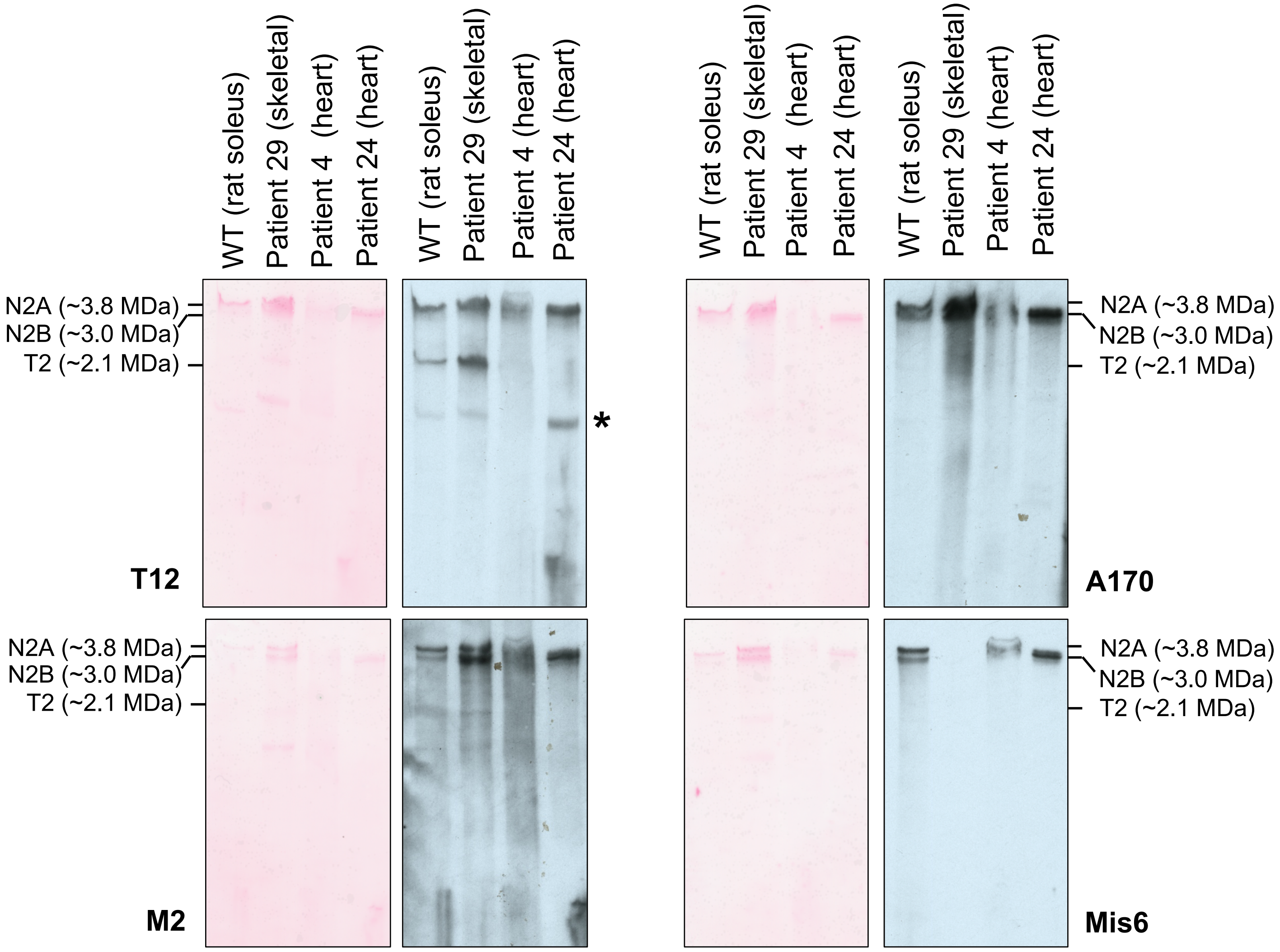

Supplementary Figure 1

Western blots of low density SDS-PAGE gels probing Titin expression in skeletal or cardiac tissue from three congenital myopathy patients. Antibodies used recognise either the N-terminal (T12) or C-terminal region (A170, TM2, Mis6) of Titin. Asterisk next to possible truncated fragment in patient 24 sample. Migration of Titin transcript N2A (skeletal muscle), N2B (cardiac muscle) and N-terminal degradation product T2 indicated.

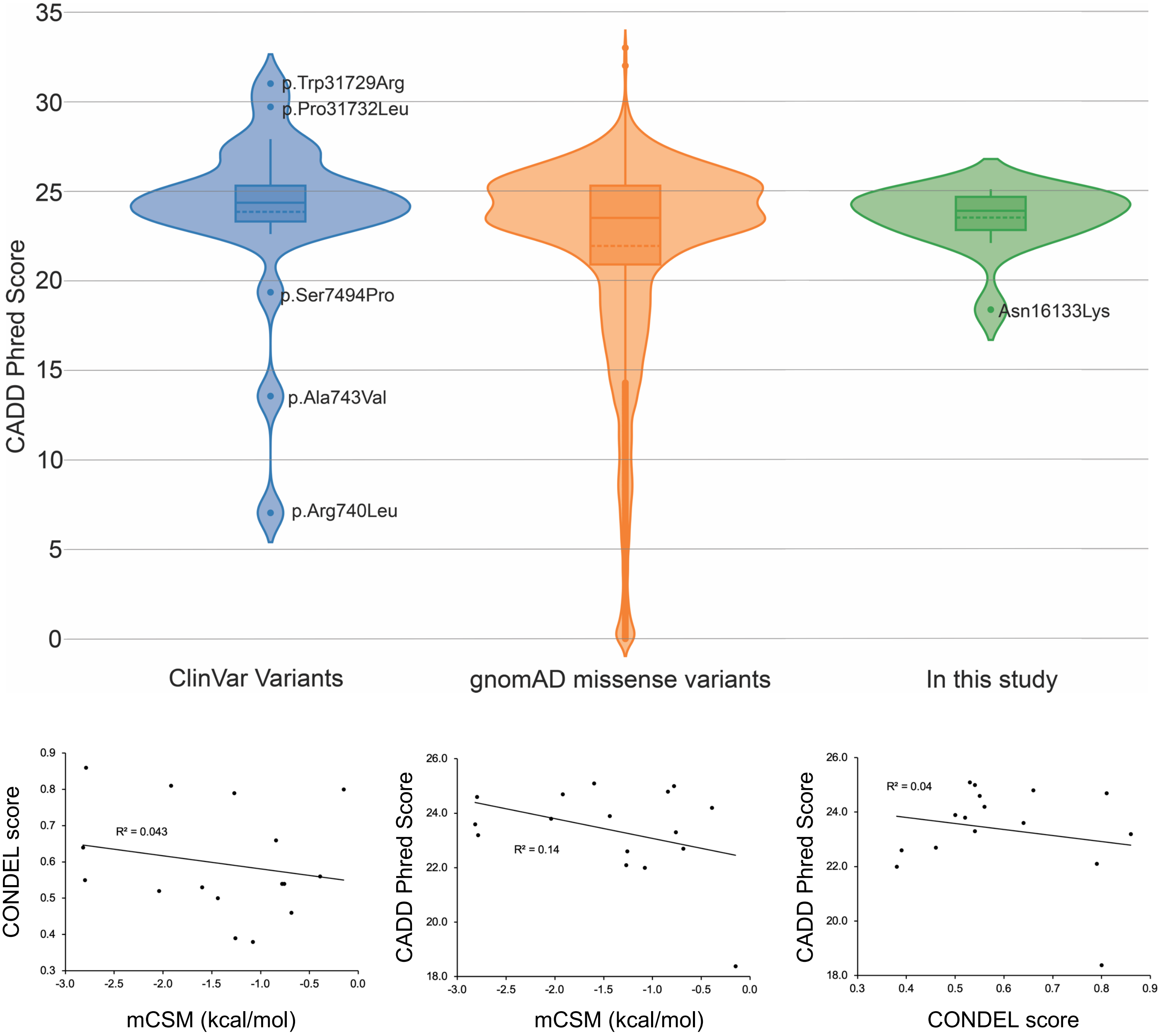

Supplementary Figure 2

Bioinformatic analysis of titin missense variants. Violin plots with statistical analysis of the CADD phred scores of reported disease causing titin missense variants from ClinVar database and selected missense variants for this study compared to all gnomAD titin missense variants (top). The Mann-Whitney U test showed a mild significance ($p=0.0412$) in the comparison of published ClinVar variants vs. gnomAD variants while the selected variants showed no significance ($p<0.7549$). Plots comparing mCSM and CONDEL values and CADD Phred scores for missense variants in the disease cohort (bottom).

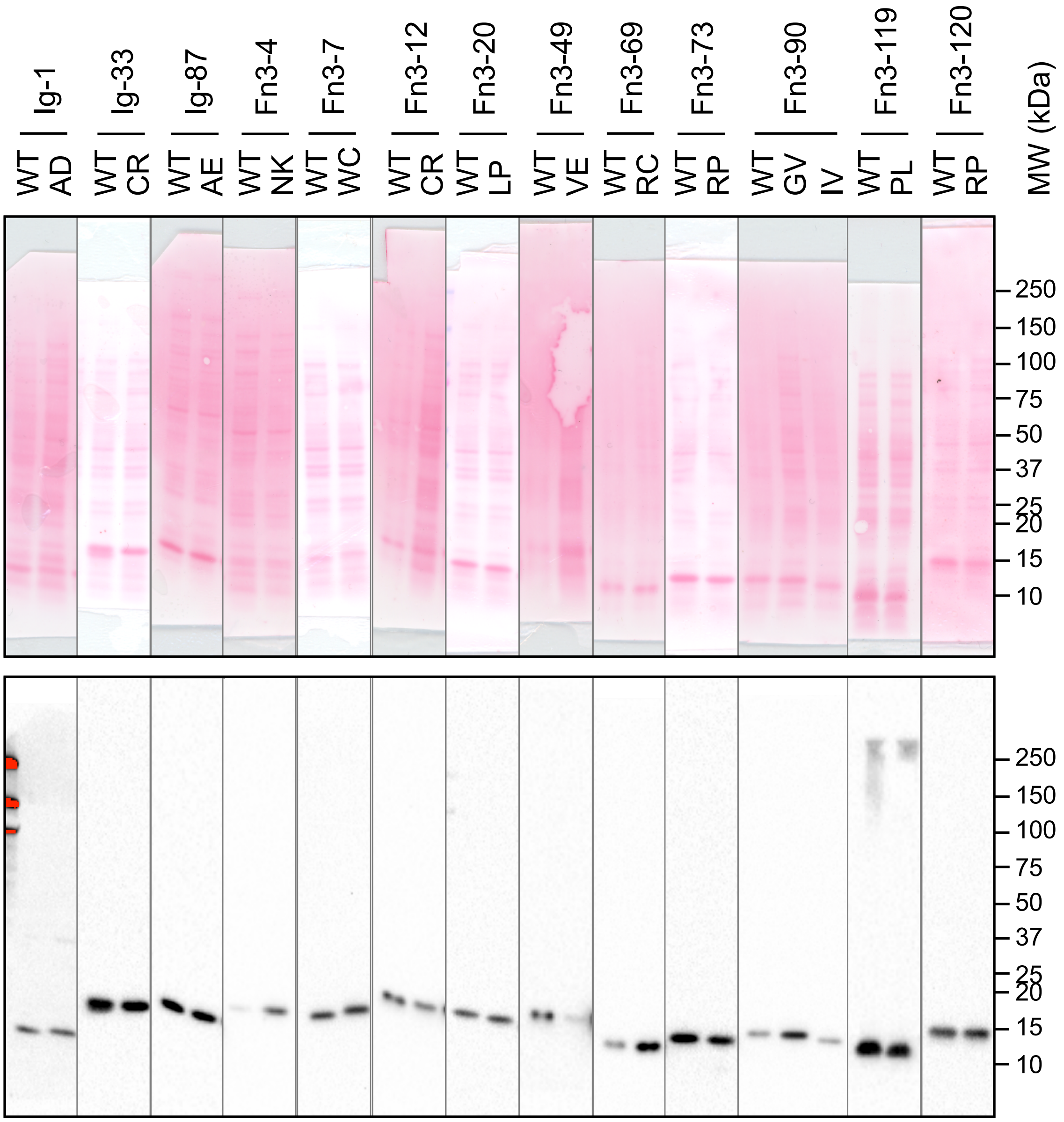

Supplementary Figure 3

Western blot assessing total expression of WT and missense variant-containing titin domains. Bacterial cultures expressing His-tagged titin domains were lysed, separated into total and soluble fractions, run on Western blot and probed with anti-His tag antibody. Total fractions including Ponceau red staining shown here without cropping.

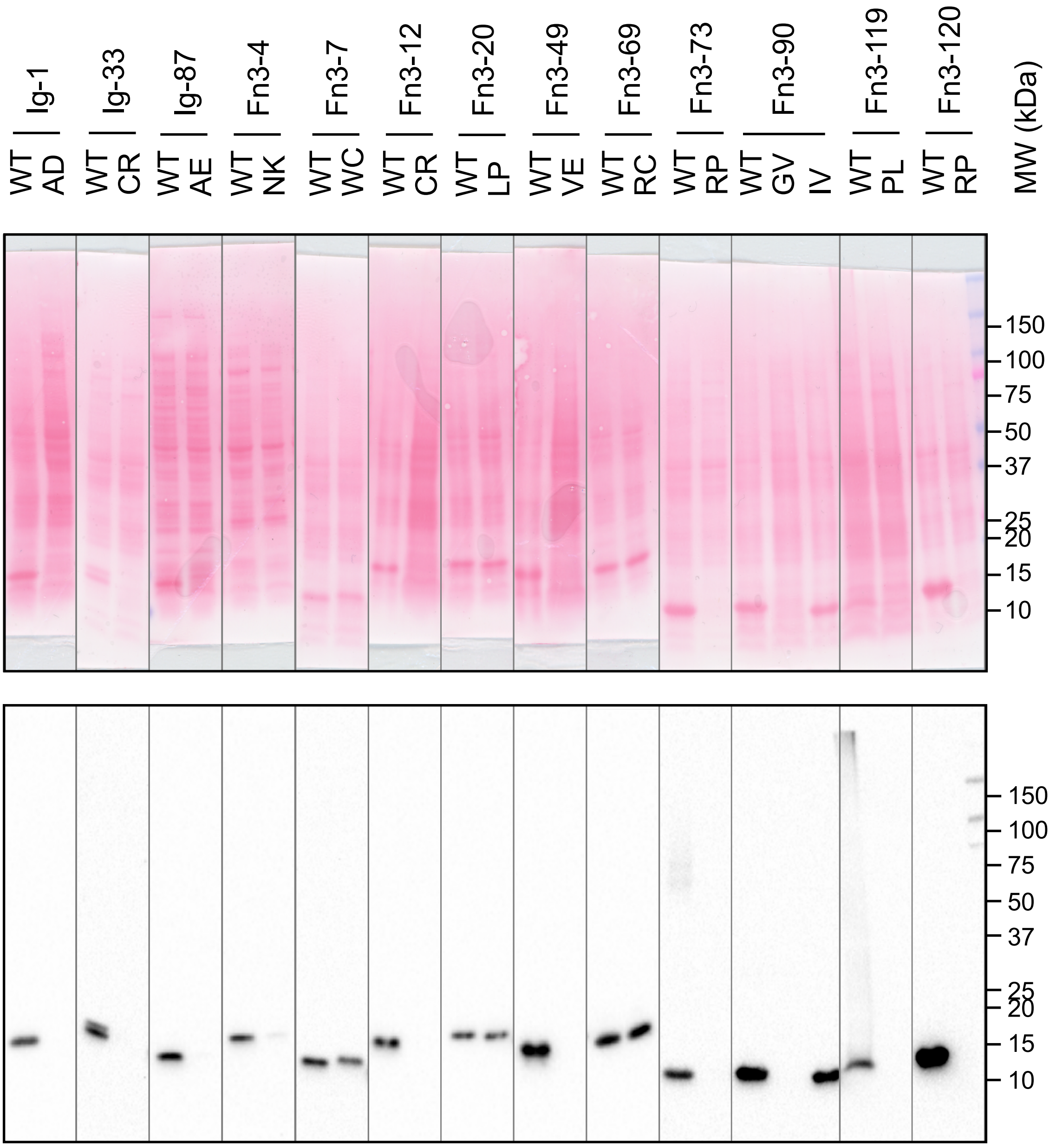

Supplementary Figure 4
Western blot assessing soluble expression of WT and missense variant-containing titin domains. Soluble fractions including Ponceau red staining shown here without cropping.

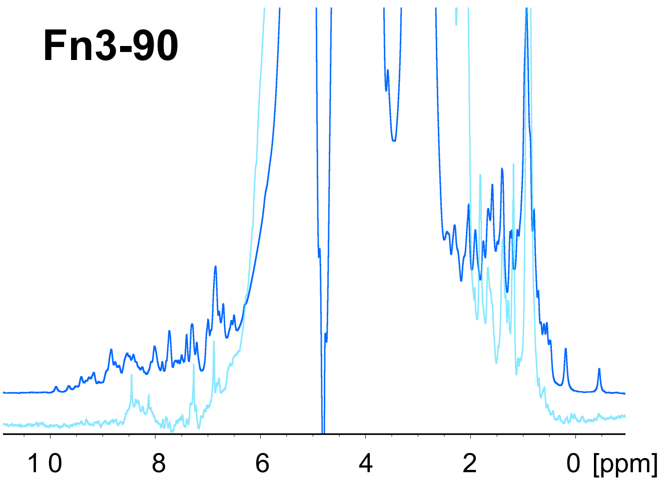

Supplementary Figure 5
1D nuclear magnetic resonance of Fn3-90 WT (dark blue) and Gly27849Val (light blue).

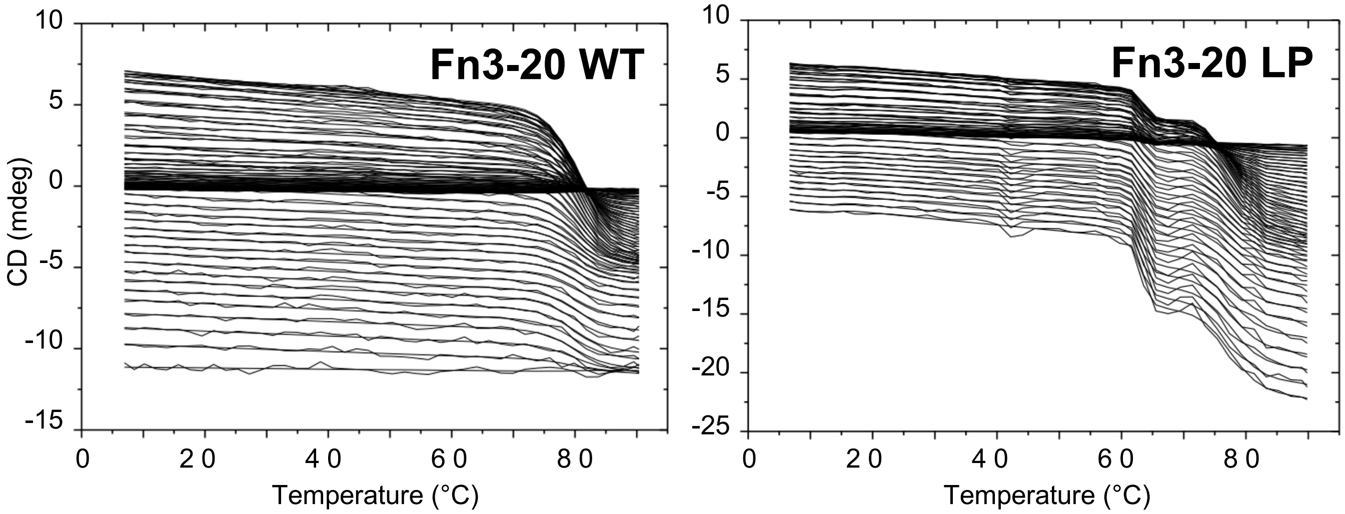

Supplementary Figure 6
Thermal denaturation circular dichroism of Fn3-20 WT (left) and Leu18237Pro (right).

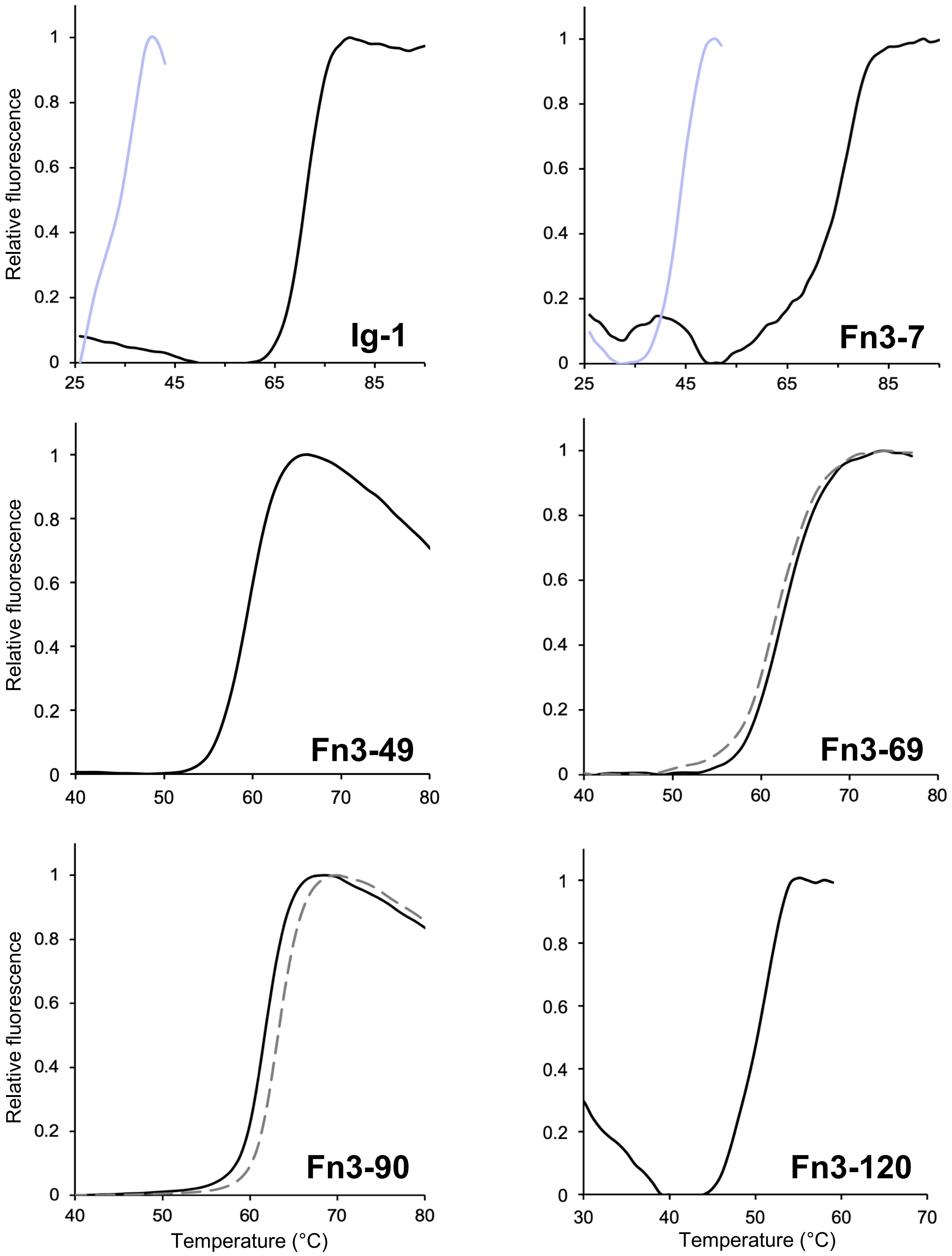

Supplementary Figure 7
Differential scanning fluorimetry thermal denaturation curves of selected WT and missense variant-containing titin domains. Shown here are denaturation curves for WT and disease cohort variants Ig-1 WT and Ala82Asp, Fn3-7 WT and Trp16471Cys, Fn3-49 WT, Fn3-69 WT and the common variant Arg24947Cys, Fn3-90 WT and the common variant Ile27775Val, and Fn3-120 WT. WT domain denaturation curves are shown in black, missense variant domain curves are shown in grey. No denaturation curves were observed for Fn3-49 Val22232Glu, Fn3-90 Gly27849Val and Fn3-120 Arg31847Pro.

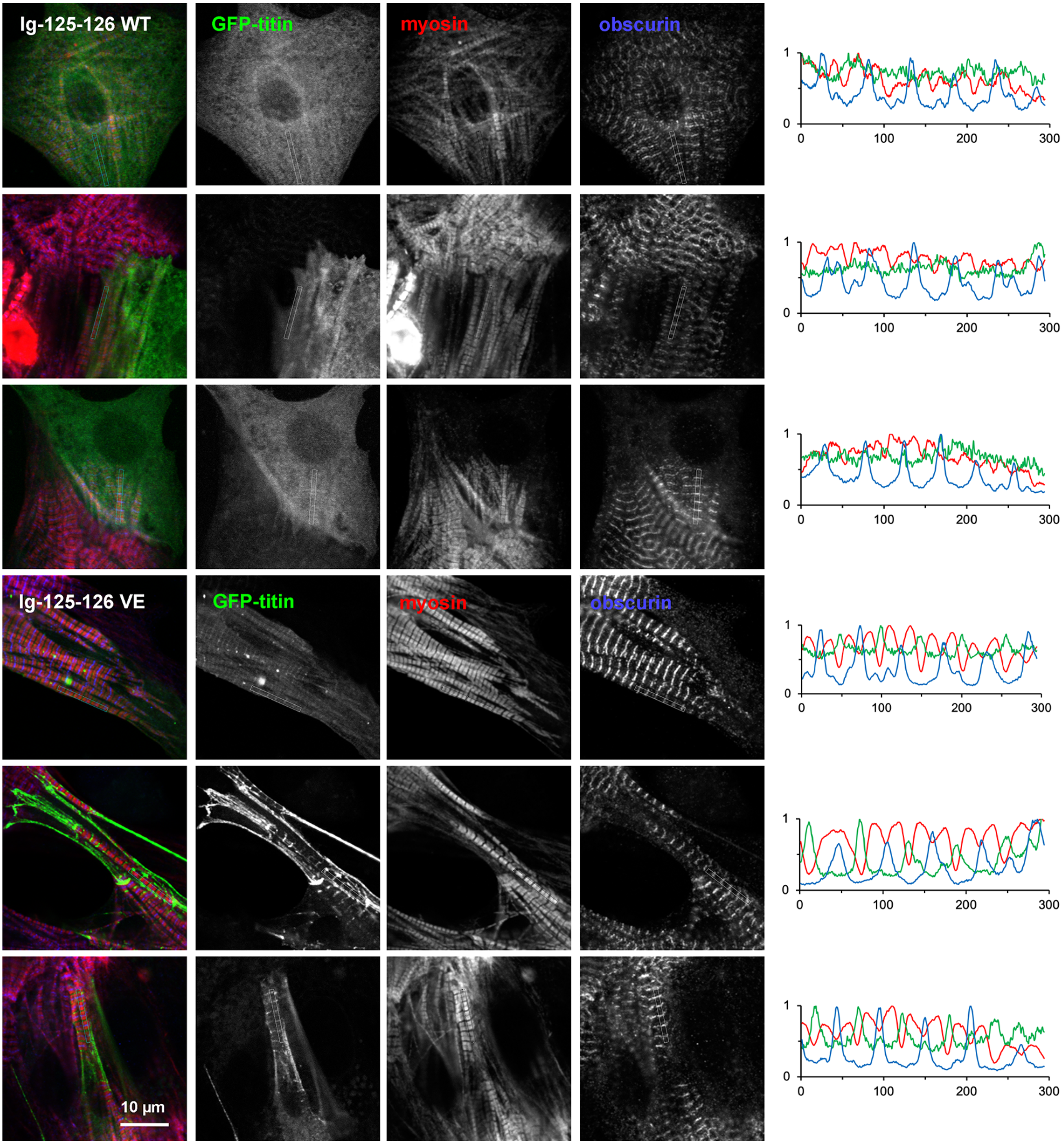

Ig-125-126 WT
GFP-titin
myosin
obscurin
Ig-125-126 VE
GFP-titin
myosin
obscurin
10 µm
1
0
0
100
200
300

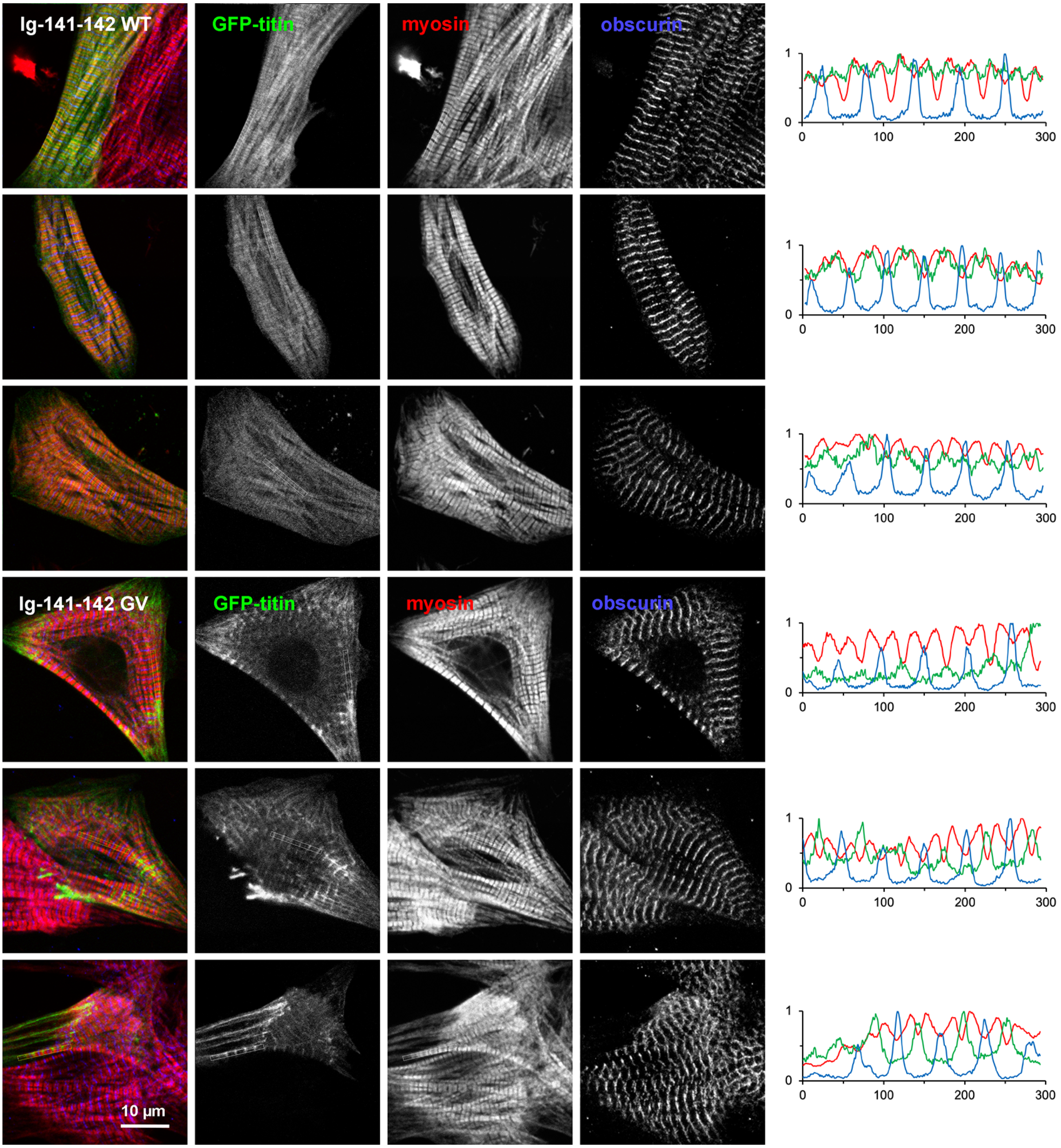

Supplementary Figure 8 (this and previous page)

Confocal immunofluorescence microscopy of neonatal rat cardiomyocytes expressing GFP-tagged titin Ig-125-126 WT and Val22232Glu, and Ig141-142 WT and Gly27849Val. Line scans of the boxed region show the localization of GFP-titin (green), myosin (red) and obscurin (blue).

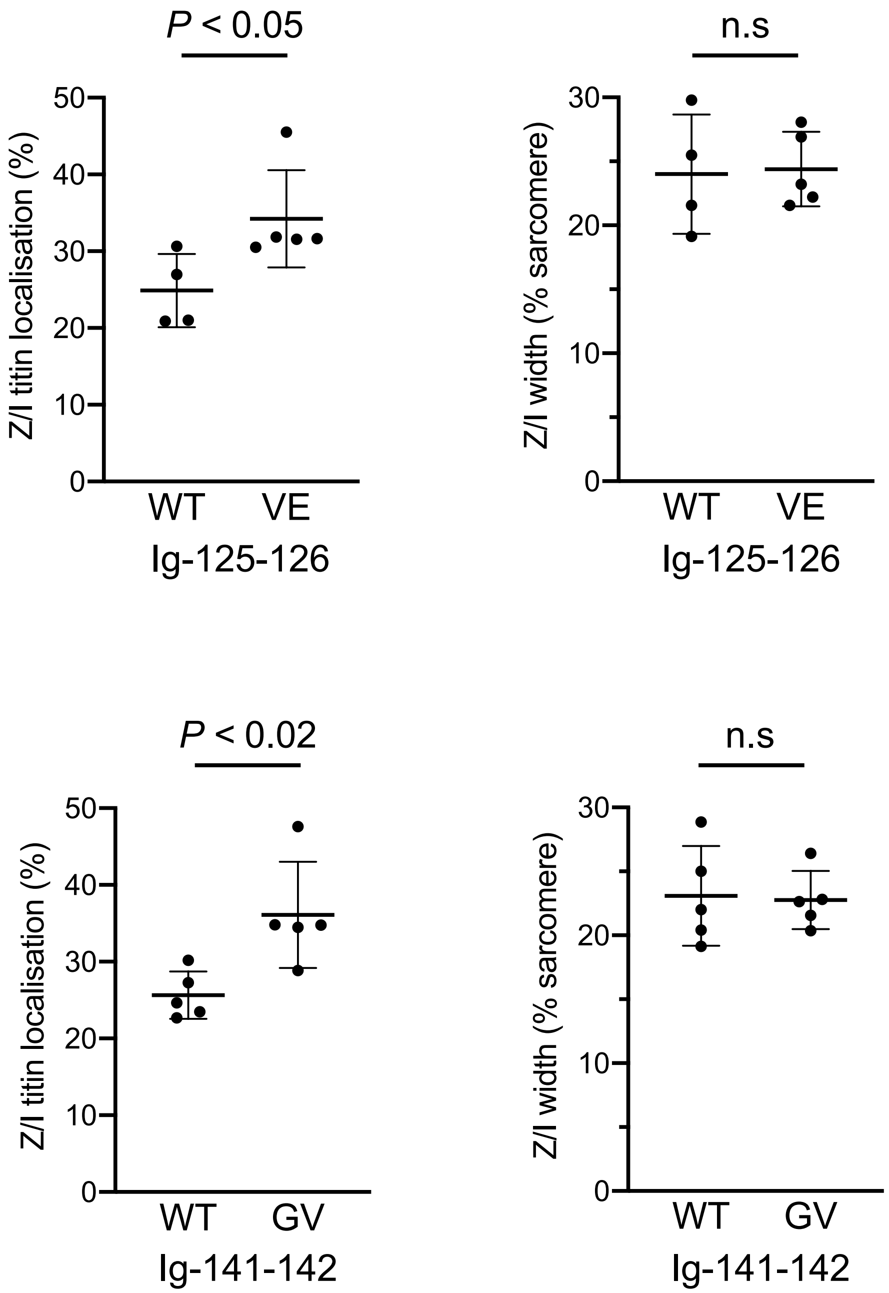

Supplementary Figure 9

Quantification of titin fragment localization to the Z-disk / I-band. The amount of expressed titin fragments Ig-125-126 and Ig-141-142, and their variants Val22232Glu and Gly27849Val, respectively, that localized to the I-band / Z-disk region was quantified as a percentage of total GFP-titin signal in 5 different cells. The increase in GFP-titin signal in the I-band / Z-disk region in the cells expressing the variant titin fragments cannot be explained by an increase in the I-band / Z-disk region width. The students t-test was performed to measure differences between the groups. Mean and standard deviation plotted.

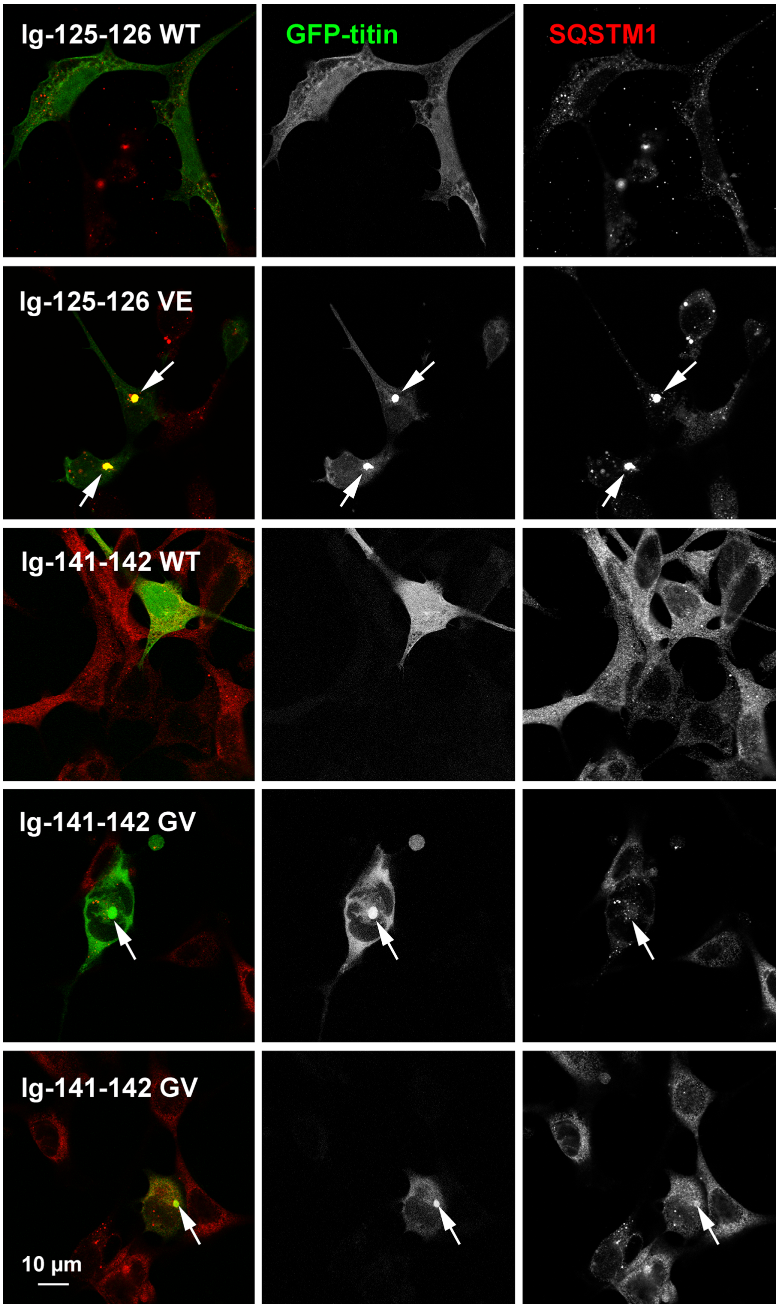

Supplementary figure 10

Confocal microscopic images showing expression of titin fragments in HEK293 cells. GFP-tagged WT titin fragments Ig-125-126 and Ig-141-142, and their variants Val22232Glu and Gly27849Val, respectively, were transfected into HEK293 cells and their expression assessed. The localisations of the titin fragments were classified into three groups: diffuse expression, speckles without colocalization with SQSTM1 and speckles with colocalization with SQSTM1. Images shown here are examples from the most common classification for each titin fragment (Ig-125-126 WT: diffuse; Ig-125-126 VE: speckles colocalized with SQSTM1; Ig-141-142 WT: diffuse), with an example of Ig-141-142 Gly27849Val from speckles without colocalization with SQSTM1 and speckles with colocalization.

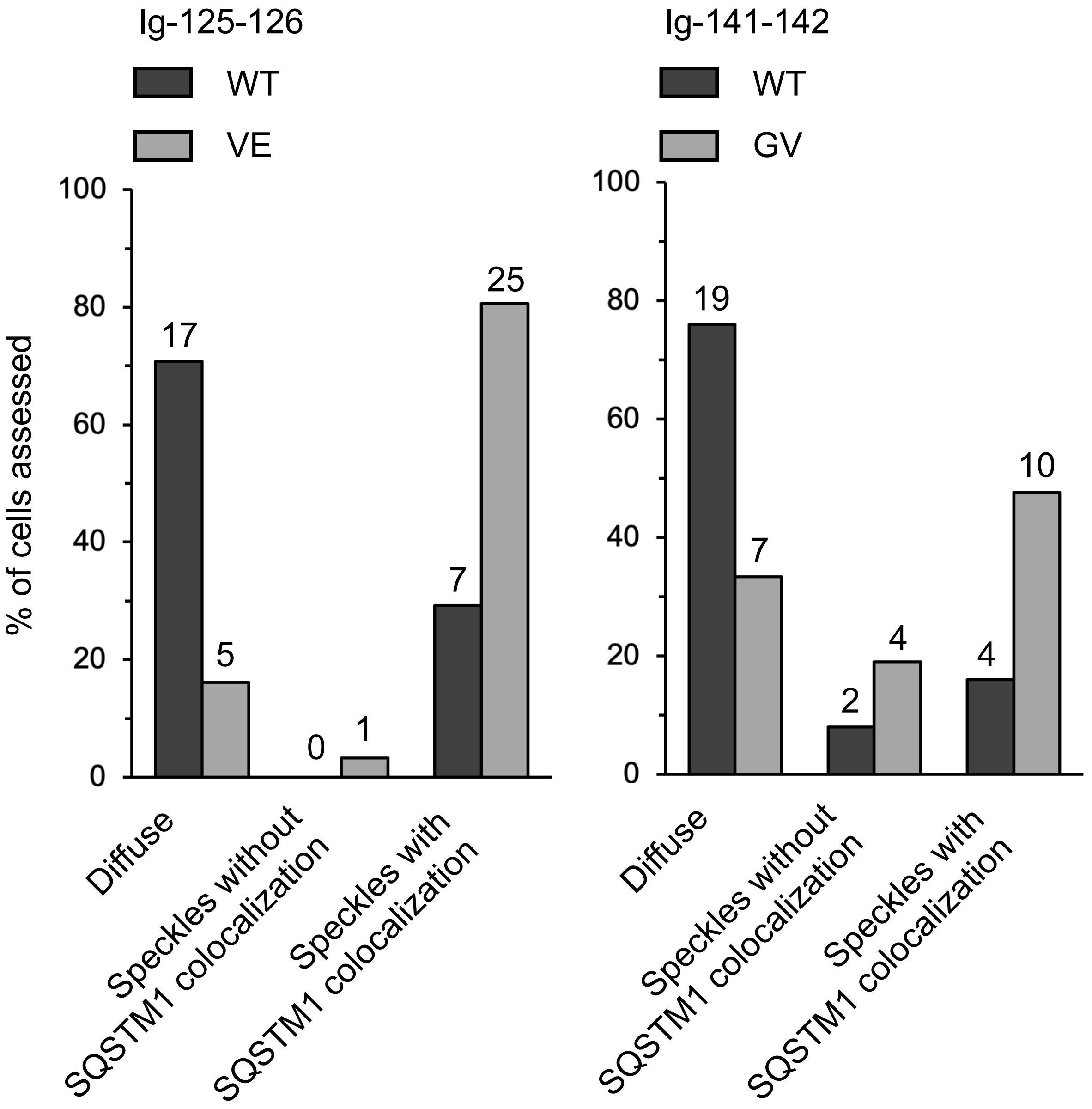

Supplementary Figure 11
Bar chart showing classification of localisation of titin fragments expressed in HEK293 cells. Cell counts shown above each bar.

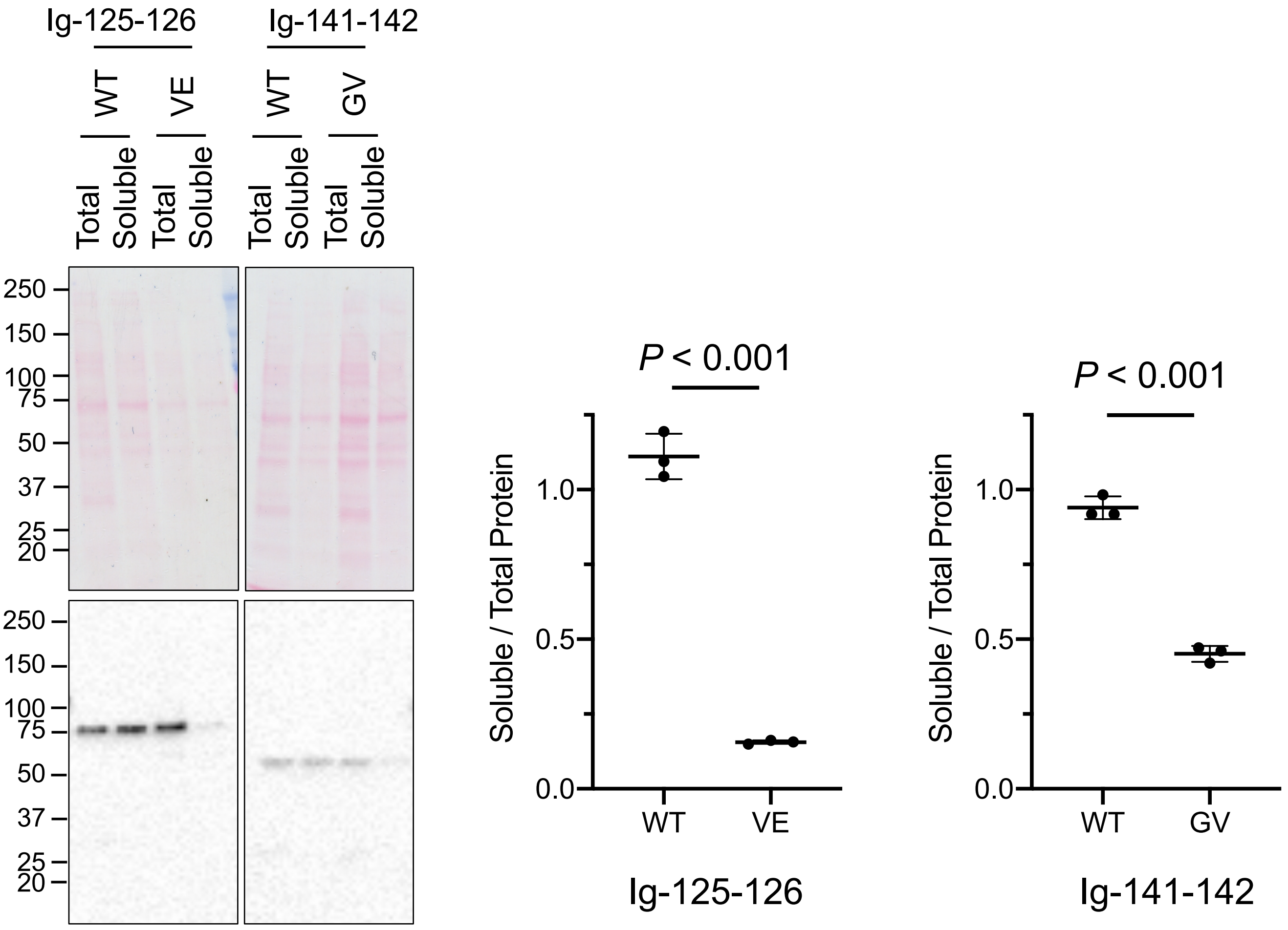

Supplementary Figure 12

Assessment of solubility of WT and variant titin fragments expressed in HEK293 cells. Left: Western blot of total and soluble fractions of HEK293 cells expressing GFP-tagged multidomain WT titin fragments Ig-125-126 and Ig-141-142, and their Val22232Glu (Fn3-49) and Gly27849Val (Fn3-90) variants, respectively, probed with anti-GFP antibody. Right: quantification of anti-GFP band density from three Western blots of three transfections of HEK293 cells with GFP-tagged multidomain titin fragments. The proportion of soluble titin may be slightly overestimated due to the smaller volume of the soluble fraction compared to the total sample. The students t-test was performed to measure differences between the groups. Mean and standard deviation plotted.

Domain	DNA		Protein	
	Start	Finish	Start	Finish
**Ig-1**	226	525	1	100
**Ig-33**	15160	15441	4979	5072
**Ig-87**	41275	41553	13684	13776
**Fn3-4**	48385	48687	16054	16154
**Fn3-7**	49570	49875	16449	16550
**Fn3-12**	51361	51660	17046	17145
**Fn3-20**	54718	55038	18165	18271
**Ig-125-126**	65575	67062	21859	22354
**Fn3-49**	66679	66996	22152	22257
**Fn3-69**	74944	75249	24907	25008
**Fn3-73**	76435	76743	25404	25506
**Ig-141-142**	83236	84355	27671	28055
**Fn3-90**	83518	83820	27765	27865
**Fn3-119**	95344	95643	31707	31806
**Fn3-120**	95644	95949	31807	31908

Supplementary Table 1
DNA and protein phasing for domains expressed in this study, corresponding to the Titin IC transcript (NM_001267550).

Patient	Missense variant	Region	Domain	mCSM (kcal/mol)	CONDEL	CADD Phred	GnomAD MAF
**1**	p.Ala82Asp	Z-disk	Ig-1	-2.79	0.86 (D)	23.2	N/A
**8**	p.Cys5054Arg	I-band	Ig-33	-1.27	0.79 (D)	22.1	N/A
**15 & 16**	p.Ala13715Glu	I-band	Ig-87	-0.76	0.54 (D)	23.3	N/A
**17**	p.Asn16133Lys	A-band	Fn3-4	-0.15	0.80 (D)	18.4	N/A
**18 & 19**	p.Trp16471Cys	A-band	Fn3-7	-1.92	0.81 (D)	24.7	0.000400
**20 & 21**	p.Cys17051Arg	A-band	Fn3-12	-1.44	0.50 (N)	23.9	0.000004
**22**	p.Val22232Glu	A-band	Fn3-49	-2.82	0.64 (D)	23.6	N/A
**23**	p.Arg25480Pro	A-band	Fn3-73	-0.78	0.54 (D)	25.0	N/A
**24**	p.Gly27849Val	A-band	Fn3-90	-0.39	0.56 (D)	24.2	N/A
**25 & 26**	p.Pro31732Leu	A-band	Fn3-119	-0.84	0.66 (D)	24.8	0.000012
**27**	p.Arg31847Pro	A-band	Fn3-120	-1.60	0.53 (D)	25.1	N/A
**29**	p.Leu18237Pro	A-band	Fn3-20	-0.69	0.46 (N)	22.7	0.000633
**30**	p.Trp34072Arg	A / M Junction	Kinase	-2.80	0.55 (D)	24.6	0.000008
Common Variants	p.Arg24947Cys	A-band	Fn3-69	-2.04	0.52 (D)	23.8	0.171451
	p.Ile27775Val	A-band	Fn3-90	-1.08	0.38 (N)	22.0	0.352009

Supplementary Table 4

***In silico* predictions for the effect of patient and common missense mutations.** mCSM calculates the predicted change in domain stability, with a change of below 0 to -2kCal/mol defined as destabilising and less than -2kCal/mol defined as highly destabilising. Positive values are defined as stabilising. A Condel score below 0.5 is classed as non-deleterious, above 0.5 classed as deleterious. The higher the CADD Phred score, the more deleterious the variant is predicted to be. Scores above 10 are the top 10% most deleterious variants in the genome, above 20 the most 1% deleterious etc.
